# Characterization of Post-Translational Modifications and Cytotoxic Properties of the Adenylate-Cyclase Hemolysin Produced by Various *Bordetella pertussis* and *Bordetella parapertussis* Isolates

**DOI:** 10.3390/toxins9100304

**Published:** 2017-09-26

**Authors:** Valérie Bouchez, Thibaut Douché, Mélody Dazas, Sophie Delaplane, Mariette Matondo, Julia Chamot-Rooke, Nicole Guiso

**Affiliations:** 1 Institut Pasteur, Unité Prévention et Thérapie Moléculaires des Maladies Humaines, 25 rue du Dr Roux, 75724 Paris, CEDEX 15, France; melody.dazas@pasteur.fr (M.D.); sophie.delaplane@yahoo.fr (S.D.); nicole.guiso@pasteur.fr (N.G.); 2 Institut Pasteur, Unité de Spectrométrie de Masse pour la Biologie, CNRS/Institut Pasteur USR2000, CITECH, 28 rue du Dr Roux, 75724 Paris, CEDEX 15, France; thibaut.douche@pasteur.fr (T.D.); mariette.matondo@pasteur.fr (M.M.); julia.chamot-rooke@pasteur.fr (J.C.-R.)

**Keywords:** adenylate cyclase hemolysin, *Bordetella pertussis*, *Bordetella parapertussis*, PTMs

## Abstract

*Bordetella pertussis* and *Bordetella parapertussis* are the causal agents of whooping cough in humans. They produce diverse virulence factors, including adenylate cyclase-hemolysin (AC-Hly), a secreted toxin of the repeat in toxins (RTX) family with cyclase, pore-forming, and hemolytic activities. Post-translational modifications (PTMs) are essential for the biological activities of the toxin produced by *B. pertussis*. In this study, we compared AC-Hly toxins from various clinical isolates of *B. pertussis* and *B. parapertussis*, focusing on (i) the genomic sequences of *cyaA* genes, (ii) the PTMs of partially purified AC-Hly, and (iii) the cytotoxic activity of the various AC-Hly toxins. The genes encoding the AC-Hly toxins of *B. pertussis* and *B. parapertussis* displayed very limited polymorphism in each species. Most of the sequence differences between the two species were found in the C-terminal part of the protein. Both toxins harbored PTMs, mostly corresponding to palmitoylations of the lysine 860 residue and palmoylations and myristoylations of lysine 983 for *B. pertussis* and AC-Hly and palmitoylations of lysine 894 and myristoylations of lysine 1017 for *B. parapertussis* AC-Hly. Purified AC-Hly from *B. pertussis* was cytotoxic to macrophages, whereas that from *B. parapertussis* was not.

## 1. Introduction

Whooping cough is a vaccine-preventable disease caused by *Bordetella pertussis* or *Bordetella parapertussis* in humans. Both these pathogens produce many virulence factors [1]: adhesins, such as filamentous hemagglutinin (FHA) and pertactin (PRN), and toxins, such as pertussis toxin (PT), which is produced specifically by *B. pertussis*, and adenylate cyclase hemolysin (AC-Hly). As for other *Bordetella* virulence factors [2], the expression of these factors is regulated by Bvg [2]. Moreover, as recently shown in *B. pertussis*, the expression of PT and AC-Hly is also modulated by RpoE sigma factor [3]. *B. pertussis* AC-Hly (Bp-AC-Hly) is a 1706-amino acid protein (Uniprot P0DKX7) with an adenylate cyclase (AC) domain in its first 400 amino acids and a 1306-amino acid repeat in toxins (RTX) domain consisting of hydrophobic pore-forming (500–700), fatty acyl-modified (800–1000), calcium-binding (1000–1600), and C-terminal secretion signal subdomains. The AC-Hly of *B. parapertussis* (Bpp-AC-Hly) has a similar structure [4,5]. These proteins are encoded by the *cyaA* gene (BP0760 in *B. pertussis* and BPP0321 in *B. parapertussis*).

The Bp-AC-Hly protein has a complex structure and multiple activities [6,7,8,9]: (i) binding to the host cell, (ii) translocation of the enzymatic domain (AC), resulting in supraphysiological intracellular cyclic adenosine monophosphate (cAMP) levels after calmodulin activation, and (iii) Ca^2+^-dependent pore-forming activity on target cells [10,11]. Skopova et al. [12] recently used different *B. pertussis* AC-Hly mutants to demonstrate that the ability of Bp-AC-Hly to increase cAMP levels was sufficient for lung infection, but both AC and pore-forming activities were required for full virulence. Bp-AC-Hly and Bpp-AC-Hly must undergo post-translational modifications (PTMs) for activity. These PTMs are mediated by an acyl transferase encoded by the *cyaC* gene, which is co-expressed with *cyaA* [5]. Two PTM sites have been identified in Bp-AC-Hly: the lysine residues in positions 860 and 983 [13,14,15,16] according to the PTM database (http://dbptm.mbc.nctu.edu.tw). The lysine 983 residue of Bp-AC-Hly is palmitoylated, but the nature of the modification to this residue in Bpp-AC-Hly is unknown. Both Bp- and Bpp-AC-Hly induce protective immunity in the murine respiratory model [17,18,19,20]. 

AC-Hly is difficult to purify from *B. pertussis* cultures. Moreover, Bp-AC-Hly is a hydrophobic protein that aggregates or is degraded during purification. Its solubilization requires a denaturing agent, such as urea. For these reasons, most structural, biological, and immunological studies of Bp-AC-Hly have used the recombinant Bp-AC-Hly. This molecule is produced in *E. coli* K-12 harboring the *B. pertussis* 18323-*cyaA* and 18323-*cyaC* genes, encoding the toxin and the enzyme required to the appropriate modifications on the toxin, respectively [21]. However, the *B. pertussis* 18323 strain belongs to a different genomic clade from the other *B. pertussis* isolates circulating around the world [22,23]. In addition, the recombinant Bp-AC-Hly produced in *E. coli* harbors different PTMs and has different biological and protective activities from those of the toxin produced directly by *B. pertussis* isolates [14,24]. 

Over the last four decades, *B. pertussis* and *B. parapertussis* genomes have been shown to evolve under vaccination- and disease-induced pressure, adapting to human populations [22,23,25,26,27,28]. The genomes of *B. pertussis* and *B. parapertussis* contain different insertion sequences, mostly IS*481* for *B. pertussis* and IS*100*1 for *B. parapertussis*, and these sequences make a major contribution to the deletion, insertion, or inactivation of genes, as observed in some *B. pertussis* isolates displaying IS*481* insertions within the *prn* gene [29]. The gene encoding Bp-AC-Hly has never been deleted or inactivated in any *B. pertussis* or *B. parapertussis* isolates, whereas the corresponding Bb-AC-Hly *cyaA* gene of some *B. bronchiseptica* isolates can be replaced with a peptide transport protein operon [23,30]. Only one clinical isolate of *B. pertussis* presenting a duplication of *cyaA* has been described to date [31].

Given the important role of AC-Hly in the pathogenesis of *B. pertussis*, many experts around the world have proposed the inclusion of recombinant AC-Hly in pertussis acellular vaccines [8]. In this context, we felt that it was important (i) to compare the *B. pertussis* and *B. parapertussis cyaA* genes; (ii) to compare the PTMs of the AC-Hly toxins produced by various *B. pertussis* isolates with different properties; (iii) to characterize and compare the PTMs of Bpp-AC-Hly toxins produced by various *B. parapertussis* isolates; and (iv) to compare the cytotoxic activities of Bp- and Bpp-AC-Hly.

## 2. Results

### 2.1. Genomic Analysis of the B. pertussis and B. parapertussis cyaA Genes

We analyzed polymorphism of the *cyaA* gene using the *Bordetella* virulence-associated genes scheme of the *Bordetella* MLST database and considering the locus BORD005031 corresponding to *cyaA* (https://pubmlst.org/bordetella/). This locus has 47 alleles in the various *Bordetella* species producing AC-Hly for which data are recorded in the database: 35 alleles from *B. bronchiseptica*, 3 from *B. parapertussis* (alleles 3, 5, and 37), and 9 from *B. pertussis* (alleles 1, 4, 7, 8, 9, 42, 43, 44, and 47). Figure S1 presents the polymorphisms of the 47 different *cyaA* alleles.

Focusing on the nine alleles from *B. pertussis* and considering the 603 isolates for which data are publicly available for this locus in the database, we found that 90.0% of the isolates (i.e., 543/603) had allele 4, as in the Tohama reference strain; 7.6% (i.e., 41/603) had allele 7, as in the 18323 reference strain; 1.5% (i.e., 9/603) had allele 9; 0.9% (i.e., 6/603) had allele 42; and the remaining alleles (8, 43, 44, and 47) were each present in a single isolate (1/603). Four of these nine alleles are synonymous and five encode proteins with amino-acid substitutions. Non-synonymous alleles are nevertheless infrequent, because such alleles were found in only 2.9% (18/603) of isolates, most of which were collected between 1960 and 1995. Data for only five isolates of *B. parapertussis* were publicly available from the *Bordetella* MLST database, including the human reference strain Bpp12822 and the ovine reference strain Bpp5. Only three different alleles were identified for *B. parapertussis*: two in human isolates (alleles 3 and 5) and one in an ovine isolate (allele 37). We extended this analysis to recently obtained human *B. parapertussis* isolates, which were also all found to contain allele 3, illustrating the high degree of conservation of the *cyaA* gene in *B. parapertussis* isolates (data not shown). We found that two polymorphic sites resulting in amino-acid substitutions were specific to allele 37 of the ovine Bpp5 (M37V and S560A) strain, one was specific to allele 5 (T1139S), and two others were specific to human isolates (i.e., V567G and G1249D) (Figure S1). A more detailed examination of Figure S1 reveals that some of the nonsynonymous polymorphisms found in *B. parapertussis* are also common to *B. bronchiseptica*; there are four such polymorphisms in the sequence encoding amino acids 800 to 1000 of the protein and 29 in the sequence encoding amino acids 1000–1600 of the protein. This is in agreement with previous reports [4,9,32]. These findings indicate that the Bpp-*cyaA* gene is closer to Bb-*cyaA* than to Bp-*cyaA*, and that there are large differences in this part of the protein between Bp-AC-Hly and the other two proteins.

### 2.2. Partial Purification of AC-Hly

Extraction with urea and by affinity chromatography on a calmodulin column resulted in similar purification yields for all isolates: 1.4 ± 0.4 mg/mL for all *B. pertussis* isolates. Similar amounts of protein (1.5 ± 0.3 mg/mL) were obtained from PRN-deficient *B. pertussis* isolates, but yields were lower for FHA-deficient *B. pertussis* isolates (0.9 ± 0.3 mg/mL). Similar amounts of protein were obtained from all *B. parapertussis* isolates (1.3 ± 0.4 mg/mL) and PRN-deficient *B. parapertussis* isolates (1.1 ± 0.1 mg/mL).

**Figure 1:**
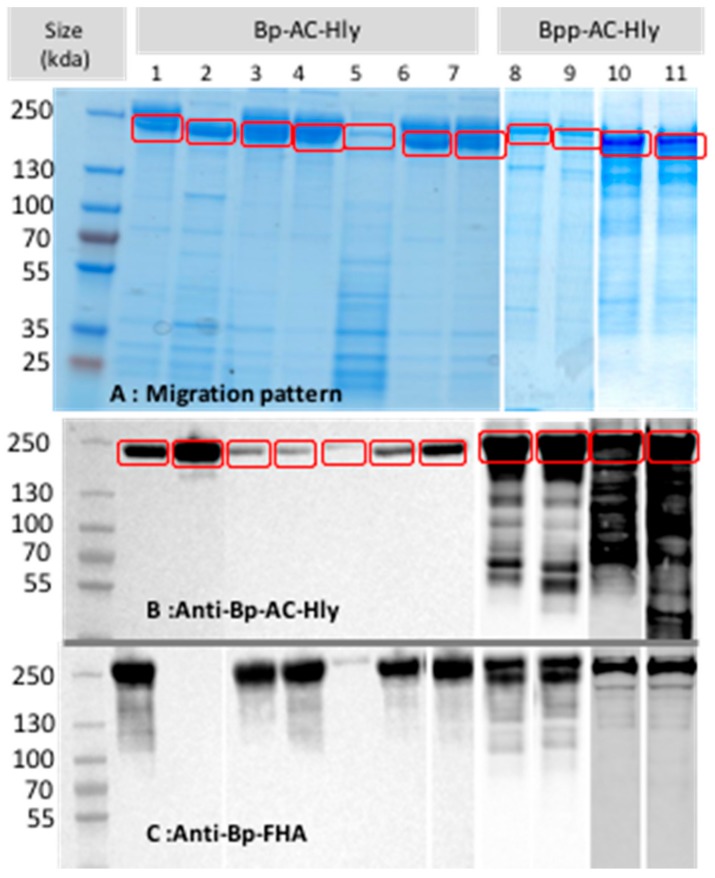
(**A**) Protein migration pattern, (**B**) Western blot with a polyclonal anti-Bp-AC-Hly antibody, (**C**) Western blot with a polyclonal anti-Bp-FHA antibody (lane 1: Tohama, lane 2: FR4624, lane 3: FR5133, lane 4: FR5187, lane 5: CIP1672, lane 6: FR5388, lane 7: FR5392, lane 8: Bpp12822, lane 9: BPP1, lane 10: FR3728, lane 11: FR5840) Full length AC-Hly is surrounded. Bp = *B. pertussis*; Bpp = *B. parapertussis*; AC-Hly = adenylate cyclase hemolysin; and FHA = filamentous hemagglutinin.

The purified AC-Hly preparations contained several additional proteins (Figure 1A); some were proteolysis fragments, as shown by Western blotting with a polyclonal anti-AC-Hly serum (Figure 1B), whereas others were contaminants, including FHA (Figure 1C). The Bpp-AC-Hly were more proteolyzed than the Bp-AC-Hly.

### 2.3. Post-Translational Modifications

**Figure 2:**
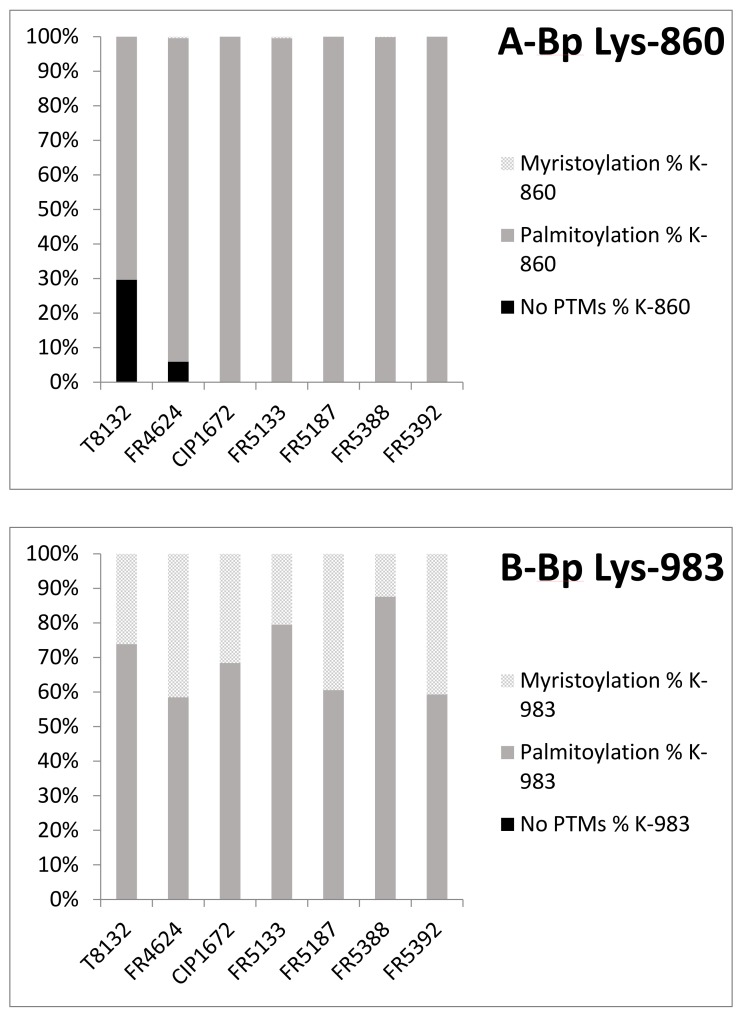
Post-translational modifications (PTMs) observed for Bp-Ac-Hly ((**A**) PTMs on K-860; (**B**) PTMs on K-983). From the modifications Specific Peptides output table of the MaxQuant results, intensities of the unmodified and modified peptides of interest (peptide forms) were extracted. For each lysine residue (K-860 and K-983), intensities were summed by peptide form and normalized against the sum of all extracted intensities per involved lysine. These calculated percentages were plotted on the bar chart representation: Myristoylation (light grey), Palmitoyalation (intermediate grey), no PTMs (dark). The intensities of peptide ions used in the bar charts (Figure 2 and Figure 3) do not necessarily directly correlate with the actual amount of each corresponding peptide since ionization efficiencies can vary with the presence of post-translational modifications. However, myristoylation and palmitoylation are comparable modifications that occur on the same amino acid (Lysine), and, thus, we can postulate that the ionization efficiencies of the corresponding peptides should not be very different.

A mass spectrometry analysis was done on purified Bp-AC-Hly and Bpp-AC-Hly after tryptic digestion (Fragmentation spectra are presented in Figures S2 and S3). This analysis confirmed that Bp-AC-Hly underwent post-translational modifications. The following PTMs were observed for all the purified preparations of Bp-AC-Hly: a palmitoyl group on lysine 860 and a palmitoyl or a myristate group on lysine 983 (Figure 2).

**Figure 3:**
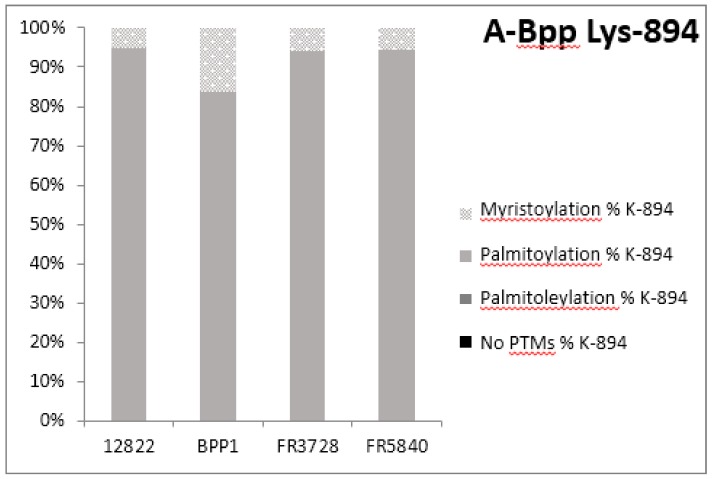

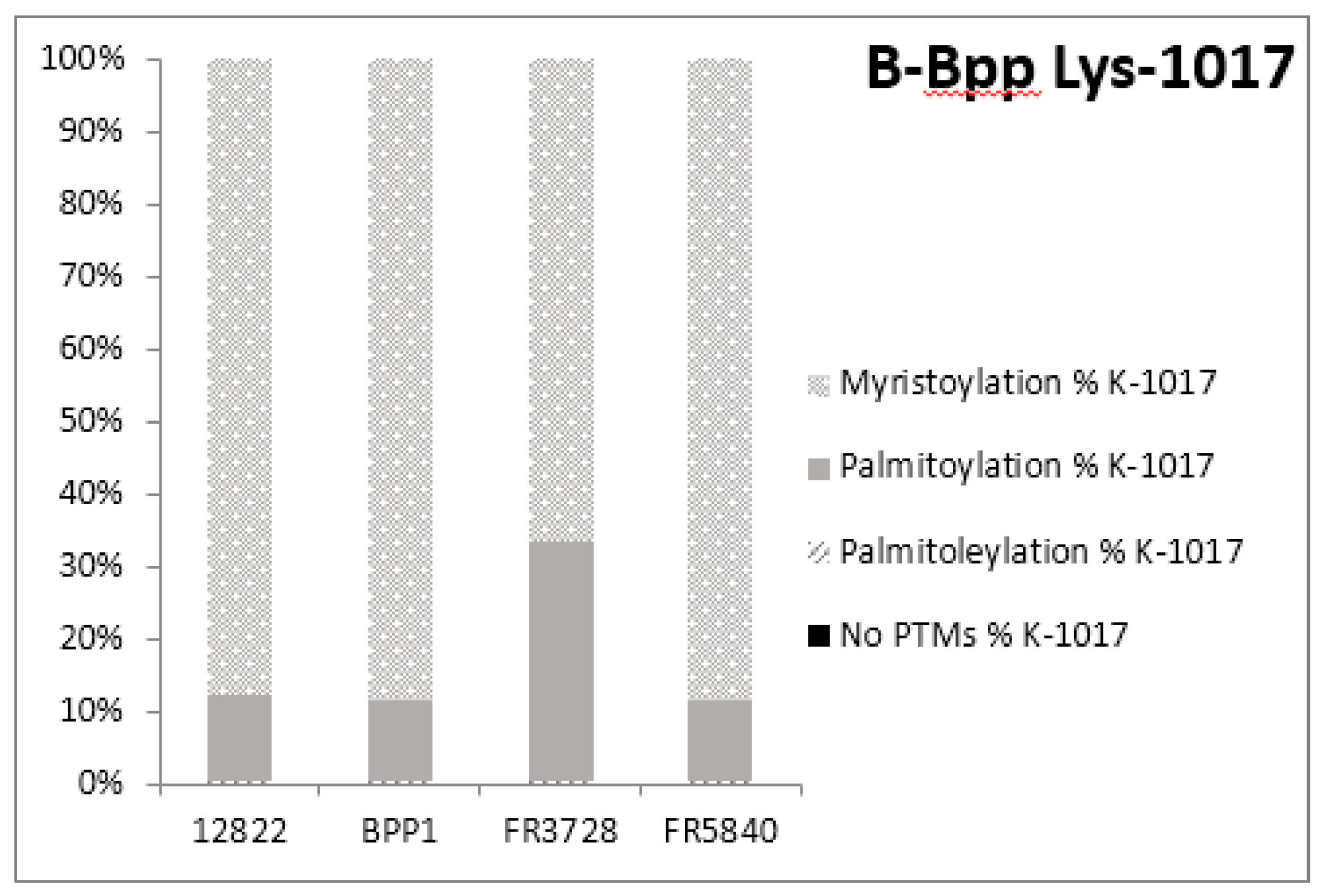
PTMs observed for Bpp-Ac-Hly ((**A**) PTMs on K-894; (**B**) PTMs on K-1017). From the modifications Specific Peptides output table of the MaxQuant results, intensities of the unmodified and modified peptides of interest (peptide forms) were extracted. For each lysine residue (K-894 and K-1017), intensities were summed by peptide form and normalized against the sum of all extracted intensities per involved lysine. These calculated percentages were plotted on the bar chart representation: Myristoylation (light grey), Palmitoyalation (intermediate grey), Palmitoleylation (hatched), no PTMs (dark). The intensities of peptide ions used in the bar charts (Figure 2 and Figure 3) do not necessarily directly correlate with the actual amount of each corresponding peptide since ionization efficiencies can vary with the presence of post-translational modifications. However, myristoylation, palmitoylation, and palmitoleylation are comparable modifications that occur on the same amino acid (Lysine), and, thus, we can postulate that the ionization efficiencies of the corresponding peptides should not be very different.

PTMs were also detected in the various purified preparations of Bpp-AC-Hly: mostly palmitoylation of lysine 894 and myristoylation on lysine 1017 (Figure 3).

### 2.4. Cytotoxicity toward Macrophages

**Figure 4:**
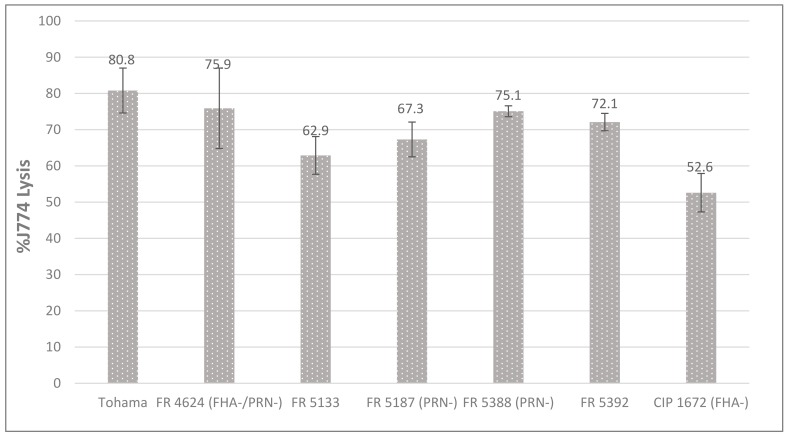
Percentage of J774.A1 cells lysed by Bp-AC-Hly. The results presented are the mean ± standard deviation of three different experiments.

We evaluated the cytotoxicity of all purified preparations of AC-Hly with J774.A1 macrophages. As shown in Figure 4 for *B. pertussis*, all Bp-AC-Hly toxins were similarly cytotoxic to these cells, whereas no cytotoxicity was observed for any of the Bpp-AC-Hly toxins (Figure S4).

## 3. Discussion

The AC-Hly protein is encoded by the *cyaA* gene (BP0760 in *B. pertussis*, BPP0321 in *B. parapertussis).* Unlike other virulence genes encoding vaccine antigen proteins displaying allelic variation (such as the *ptxA* and *prn* genes*)*, *cyaA* is stable, with few polymorphisms detected in the two species. Most *B. pertussis* isolates carried the same *cyaA* allele as the Tohama reference strain (i.e., allele 4 in the PubMLST database) and all human *B. parapertussis* isolates carried the same allele as the 12,822 reference strain (i.e., allele 3). This finding is consistent with the results of the SNP study by Bart et al. [22], which reported only two different SNPs within *cyaA*, relative to the Tohama strain, in only seven isolates (corresponding to alleles 7 and 4) from a set of more than 300 isolates collected from around the world in different vaccination eras. Our findings are also consistent with those of Chenal-Francisque et al. [32], who found no polymorphism in the C-terminal part of *cyaA* in various *B. pertussis* and *B. parapertussis* isolate*s.* However, there are a large number of sequence differences between the *cyaA* genes of *B. pertussis* and those of *B. parapertussis*, mostly affecting amino-acids 800–1000 and 1000–1600 of the protein, corresponding to the region carrying the protective epitope and receptor-binding domain sites for human cells. Based on MLST, *B. pertussis* and *B. parapertussis* belong to two different complexes that evolved recently and independently, from two different *B. bronchiseptica* complexes [28].

Despite the low purification yields for native AC-Hly, this step is a prerequisite for the study of native Bp-AC-Hly and Bpp-AC-Hly. We purified AC-Hly from cultures of various *B. pertussis* and *B. parapertussis* isolates, some of which did not produce PRN or FHA. Production and purification yields were similar for all isolates, regardless of whether they produced PRN. Smaller amounts of Bp-AC-Hly were obtained from FHA-deficient isolates which correlates with the observations of Zaretzky et al. [33]. The presence of residual FHA was detected in all Bp-AC-Hly and Bpp-AC-Hly preparations except those originating from FHA-deficient *B. pertussis* isolates. This finding is consistent with the already demonstrated strong interaction between FHA and AC-Hly: (i) FHA is thought to play a role in the surface retention of AC-Hly toxin through a physical interaction, and AC-Hly enhances the adhesin functions of FHA [33,34]; (ii) an interaction between FHA and Bp-AC-Hly was recently shown to inhibit biofilm formation in vitro [35]; (iii) the absence of FHA has been reported to lead to the enhanced expression of other virulence factors in *B. pertussis* isolates [36]; and (iv) the inhibition of AC-induced macrophage lysis by anti-FHA antibodies seems to be modified in recent isolates [37]. 

The activity of AC-Hly is dependent on PTMs. In 1994, Hackett et al. [13] demonstrated that Bp-AC-Hly (produced from the Bp338 strain, a derivative of the Tohama reference strain) displayed 100% acylation of the Lys 983 residue involving the addition of palmitoyl. The following year [14], they showed that the PTMs observed on a recombinant Bp-AC-Hly with different biological and protective activities, were not the same; the Lys 983 residue of the recombinant toxin was acylated with mainly palmitate and some miristate. Furthermore, most of the lysine 860 residues were also palmitoylated. We also detected a mixture of palmitoyl and myristoyl acylations on the lysine 983 residue of the Tohama AC-Hly, as observed for the recombinant Bp-AC-Hly, but not in the first study by Hackett et al. [13]. Myristate was also observed on the Bp-AC-Hly produced by the other isolates Furthermore, by contrast to the initial study published by Hackett [13], we observed an acylation of the lysine 860 residue in Bp-AC-Hly purified from the Tohama reference strain, as in AC-Hly from other isolates. This acylation was mostly palmitoyl, as already reported for the recombinant Bp-AC-Hly. Our preliminary results indicate that the modifications of the toxins produced by various *B. pertussis* isolates are similar to those of the recombinant toxin. These observations suggest that the differences in biological and protective activities between the recombinant toxin and the toxins produced by *B. pertussis* are not due to differences in PTMs. We suggest instead that these differences are mostly due to interactions with other components of *B. pertussis*. FHA may be one of the components involved in these interactions, possibly together with PRN and lipopolysaccharide (LPS), which have also been identified as potentially important [33,37,38,39,40,41]. The interactions between FHA and AC-Hly and those with PRN may be crucial for the final conformation of AC-Hly and for its immunogenic and protective activities. Additional studies are required to evaluate the protective activity of recombinant Bp-AC-Hly, because its conformation may be suboptimal relative to that of the Bp-AC-Hly produced and secreted by *B. pertussis*.

In this study, we found that the PTMs of Bpp-AC-Hly concerned the lysine residues in positions 894 and 1017. These modifications were essentially the palmitoylation of Lys 894, as for the Lys 860 residue of *B. pertussis*. The Lys 1017 residue was mostly myristoylated, but some palmitoylation was observed, contrary to the findings for Bp-AC-Hly. In addition, we observed that contrary to Bp-AC-Hly, none of Bpp-AC-Hly were cytotoxic for macrophages. This is in agreement with previous data obtained from direct bacteria-cell interaction cytotoxicity tests [26]. We hypothesize that the lack of cytotoxicity of Bpp-AC-Hly may reflect not only differences in PTMs, but also differences in interactions with FHA or LPS, particularly as LPS differs between *Bordetella* species [42,43,44]. In particular, the Bpp LPS contains an O-antigen, whereas the Bp LPS does not. Further studies are required to clarify this point.

In conclusion, our study confirms that AC-Hly is a toxin that is highly conserved in both *B. pertussis* and *B. parapertussis*, unlike other virulence factors, confirming its important role in the pathogenesis of whooping cough.

## 4. Materials and Methods

### 4.1. Cultures of Bacteria

We selected seven *B. pertussis* isolates and four *B. parapertussis* isolates for study, including the reference strains Tohama CIP8132 and Bpp12822. The characteristics of the isolates are presented in Table 1.

All isolates were grown on Bordet Gengou agar (BGA) (Difco, Franklin Lakes, NJ, USA,) supplemented with 15% defibrinated sheep blood, at 36 °C for 72 h. Isolates were re-plated and incubated for another 24 h before use in liquid culture and AC-Hly purification or cytotoxicity assays, as previously described [37].

### 4.2. AC-Hly Purification

After growth on a BGA plate and a first liquid preculture in Stainer and Scholte medium [46], isolates were further subcultured in 8 × 400 mL of medium, until their optical density increased from an OD_650_ 0.2 to an OD_650_ 1 ± 0.2. Cultures were then centrifugated at 9000× *g* for 25 min at 4 °C. The bacterial pellet was weighed, suspended in a Tris _50mM_/CaCl_2 0,22mM_ − Hepes_20mM_ − Urea _5M_/pH = 8 buffer (using 5 mL/g of pellet) on ice and incubated with continuous shaking at +4 °C for 1 h. The suspension was then centrifuged at 17,000× *g* for 30 min at 4 °C. AC-Hly was purified on calmodulin agarose, as previously described [47]. Eluates were concentrated with Amicon Ultra-15 and Ultra-2 devices (Merck Millipore, Billerica, MA, USA). Protein concentration was determined with a Qubit 3.0 Fluorometer (Life technologies, Carlsbad, CA, USA). Purification yields are expressed as mg/mL of concentrated eluate.

### 4.3. Western Blots

Western blot analyses were performed as previously described [48], with specific polyclonal antibodies directed against highly purified recombinant Bp-AC-Hly (a gift from P. Sebo) or highly purified Bp-FHA (a gift from GSK).

### 4.4. Cytotoxicity Assays

J774.A1 murine monocyte/macrophage-like cells (ATCC ref TIB-67) were cultured and cytotoxicity assays were performed as previously described [29]. Cytotoxicity/inhibition assays were performed as described elsewhere [37].

### 4.5. PTM Determination

Each purified protein solution was adjusted to a concentration of 4.4 M urea with 50 mM Tris-HCl pH 7.6 buffer, and 20 µg of protein was reduced in 5 mM TCEP (Sigma, St. Louis, MO, USA) and alkylated in 25 mM iodoacetamide (Sigma). Proteins were consecutively digested with rLys-C (Promega, Madison, WI, USA) and Sequencing-Grade Modified Trypsin (Promega, Madison, WI, USA) in a 1:50 ratio (enzyme:protein). The digested peptides were purified on Pierce C18 Spin Columns (Thermo Fisher Scientific, Waltham, MA, USA). Peptides were eluted in 90% ACN/0.1% FA, dried in a Speedvac, and resuspended in 2% ACN/0.1% FA.

The digests were analyzed with an Ultimate 3000 nano-HPLC (Dionex, Sunnyvale, CA, USA) coupled online to an LTQ-Orbitrap Velos Mass Spectrometer (Thermo Fisher Scientific, Waltham, MA, USA) equipped with an ETD source. We loaded 0.5 μg of peptides onto a C4 trap column (Acclaim™ PepMap™ 300, 5 μm, 300 Å, 300 μm i.d. × 5 mm—Thermo Fisher Scientific, Waltham, MA, USA) and separated them on a custom-built 20 cm C4 column (5 μm particles, 300 Å pore size, ReproSil-Pur 300 C4, Dr. Maisch GmbH, Ammerbuch-Entringen, Germany) with a one-step gradient from 2.0% to 90% solvent B (80% ACN/0.1% FA) over 43 min, at a flow rate of 300 nL/min over 78 min. Mass spectrometry (MS) data were acquired with Xcalibur software in survey scan (300–1700 *m*/*z*) mode and were analyzed in the Orbitrap mass analyzer at a resolution of 60,000. Five ETD fragmentations were then analyzed in the linear ion trap. The AGC targets for MS and MS/MS scans were set to 1E6 and 5E3, respectively. The isolation width was set at 2.0 *m*/*z* and the activation time was set at 150 ms. Selected ions were dynamically excluded for 20 s. 

Raw data were analyzed with MaxQuant software version 1.5.1.2 [49] and the Andromeda search engine [50] against the *Bordetella pertussis* and *Bordetella parapertussis* UniProt databases, containing 3258 proteins and 4161 proteins, respectively. The digestion mode was set to trypsin and a maximum of two missed cleavages were allowed. N-terminal acetylation and methionine oxidation were allowed as variable modifications and cysteine carbamidomethylation as a fixed modification. For the PTM analysis, additional N-acetylated lysines (myristoyl-4H, myristoleylation, myristoylation, palmitoleylation, palmitoylation) were considered as variable modifications. The minimum peptide length was fixed at five amino acids and the required false discovery rate was set at 1% for PSMs and proteins. The main search peptide tolerance was set at 4.5 ppm and 0.5 Da for the MS/MS match tolerance. Second peptides were used to identify co-fragmentation events and to match, between runs, an accepted match time window of 0.7 min for an alignment time window of 20 min.

From the modifications SpecificPeptides output table of the MaxQuant result, we extracted the intensities of the unmodified and modified peptides of interest (peptide forms). For a given lysine residue, intensities were summed by peptide form and normalized against the sum of all extracted intensities per involved lysine. Bar charts were created with these values (Figure 2A,B and Figure 3A,B).

## Figures and Tables

**Table 1:** Characteristics of the isolates. PRN = pertactin.

Name	Species	Year of Collection	Antigen Deficiency	References
Tohama(CIP8132)	*B. pertussis*	1954	none	[29]
FR4624	*B. pertussis*	2009	FHA/PRN	[45]
1672	*B. pertussis*	1950	FHA	[37,45]
FR5133	*B. pertussis*	2012	none	[37]
FR5187	*B. pertussis*	2012	PRN	[37]
FR5388	*B. pertussis*	2012	PRN	[37]
FR5392	*B. pertussis*	2012	none	[37]
Bpp12822	*B. parapertussis*	1993	none	[26]
BPP1	*B. parapertussis*	1990	none	[26]
FR3728	*B. parapertussis*	2007	PRN	[26]
FR5840	*B. parapertussis*	2014	PRN	this study

## Supplementary Materials

The following are available online at www.mdpi.com/2072-6651/9/10/304/s1. Figure S1: Translate—aligned protein sequences BORD005031 (*cyaA*), obtained from Locus Explorer—*Bordetella* MLST locus/sequence definitions (https://pubmlst.org/bordetella/); Figure S2: Annotated MaxQuant MS/MS spectrum corresponding to the peptide containing the modified Lysines for Bp-AC-Hly; Figure S3: Annotated MaxQuant MS/MS spectrum corresponding to the peptide containing the modified Lysines for Bpp-AC-Hly; Figure S4: Percentage of J774.A1 cells lysed by Bpp-AC-Hly. The results presented are the mean ± standard deviation of different experiments.
