# Supplementary material for: Characterization of Post-Translational Modifications and Cytotoxic Properties of the Adenylate-Cyclase Hemolysin Produced by Various Bordetella pertussis and Bordetella parapertussis Isolates
Source: Toxins (Basel). 2017 Sep 26;9(10):304. doi: 10.3390/toxins9100304 (PMC5666351; doi:10.3390/toxins9100304)
Supplement: Supplementary file 1 [file toxins-09-00304-s001.zip › FigureS1.pdf]

Supplementary Figure 1: Translate - aligned protein sequences BORD005031 (cyaA), Obtained from Locus Explorer - Bordetella MLST locus/sequence definitions (https://pubmlst.org/bordetella/)

```
10        20        30        40        50        60        70        80        90       100
          ----:----|----:----|----:----|----:----|----:----|----:----|----:----|----:----|----:----|----:----|
1_1       ....................................................................................................
2_1       ....................................................................................................
3_1       ....................................................................................................
4_1       ....................................................................................................
5_1       ....................................................................................................
6_1       ................................................................................................N...
7_1       ....................................................................................................
8_1       ....................................................................................................
9_1       ....................................................................................................
10_1      ................................................................................................N...
11_1      .............................................................................................E..N...
12_1      .............................................................................................E..N...
13_1      .............................................................................................E..N...
14_1      .............................................................................................E..N...
15_1      .............................................................................................E..N...
16_1      .............................................................................................E..N...
17_1      ................................................................................................N...
18_1      ................................................................................................N...
19_1      ................................................................................................N...
20_1      ....................................................................................................
21_1      ....................................................................................................
22_1      .............................................................................................E..N...
23_1      ....................................................................................................
24_1      ....................................................................................................
25_1      ....................................................................................................
26_1      ....................................................................................................
27_1      ....................................................................................................
28_1      .............................................................................................E..N...
29_1      ....................................................................................................
30_1      ....................................................................................................
```

```
31_1       .............................................................................................E..N...
32_1       .............................................................................................E..N...
33_1       ................................................................................................N...
34_1       .............................................................................................E..N...
35_1       ................................................................................................N...
36_1       .............................................................................................E..N...
37_1       ....................................................................................................
38_1       ....................................................................................................
39_1       ....................................................................................................
40_1       ................................................................................................N...
41_1       .............................................................................................E..N...
42_1       ....................................................................................................
43_1       ....................................................................................................
44_1       ....................................................................................................
45_1       ....................................................................................................
46_1       ....................................................................................................
47_1       ....................................................................................................
Consensus  MQQSHQAGYANAADRESGIPAAVLDGIKAVAKEKNATLMFRLVNPHSTSLIAEGVATKGLGVHAKSSDWGLQAGYIPVNPNLSKLFGRAPEVIARADNDV
                  110       120       130       140       150       160       170       180       190       200
           ----:----|----:----|----:----|----:----|----:----|----:----|----:----|----:----|----:----|----:----|
1_1        ....................................................................................................
2_1        ....................................................................................................
3_1        ....................................................................................................
4_1        ....................................................................................................
5_1        ....................................................................................................
6_1        ..................................D.................................................................
7_1        ....................................................................................................
```

PubMLST Database home Contents

8_1 ....
9_1 ....
10_1 ....
11_1 ....
12_1 ....
13_1 ....
14_1 ....
15_1 ....
16_1 ....
17_1 ....
18_1 ....
19_1 ....
20_1 ....
21_1 ....
22_1 ....
23_1 ....
24_1 ....
25_1 ....
26_1 ....
27_1 ....
28_1 ....
29_1 ....
30_1 ....
31_1 ....
32_1 ....
33_1 ....
34_1 ....
35_1 ....D...
36_1 ....
37_1 ....
38_1 ....
39_1 ....
40_1 ....D...
41_1 ....

```
42_1      ....................................................................................................
43_1      ....................................................................................................
44_1      ....................................................................................................
45_1      ....................................................................................................
46_1      ....................................................................................................
47_1      ....................................................................................................
Consensus NSSLAHGHTAVDLTLSKERLDYLRQAGLVTGMADGVVASNHAGYEQFEFRVKETSDGRYAVQYRRKGGDDFEAVKVIGNAAGIPLTADIDMFAIMPHLSN
                  210       220       230       240       250       260       270       280       290       300
          ----:----|----:----|----:----|----:----|----:----|----:----|----:----|----:----|----:----|----:----|
1_1       ....................................................................................................
2_1       ....................................................................................................
3_1       ....................................................................................................
4_1       ....................................................................................................
5_1       ....................................................................................................
6_1       .................................................................E..................................
7_1       ....................................................................................................
8_1       ....................................................................................................
9_1       ....................................................................................................
10_1      ..................................P..........................................Q......................
11_1      .................................................................E...........G......................
12_1      .................................................................E...........G......................
13_1      .................................................................E..................................
14_1      .................................................................E...........G......................
15_1      .................................................................E..................................
16_1      .................................................................E...........G......................
17_1      ..................................P.................................................................
18_1      ..................................P..........................................Q......................
19_1      ..................................P..........................................Q......................
20_1      ....................................................................................................
21_1      ....................................................................................................
22_1      .................................................................E...........G......................
23_1      ....................................................................................................
24_1      ....................................................................................................
25_1      ....................................................................................................
```

```
26_1       ....................................................................................................
27_1       ....................................................................................................
28_1       .................................................................E...........G......................
29_1       ....................................................................................................
30_1       ....................................................................................................
31_1       .................................................................E...........G......................
32_1       .................................................................E..................................
33_1       .................................................................E..................................
```



```
34_1       .................................................................E..................................
35_1       .................................................................E..................................
36_1       .................................................................E...........G......................
37_1       ..............................................................................................M.....
38_1       ....................................................................................................
39_1       ....................................................................................................
40_1       .................................................................E..................................
41_1       .................................................................E..................................
42_1       ....................................................................................................
43_1       ....................................................................................................
44_1       ....................................................................................................
45_1       ........................V...........................................................................
46_1       ....................................................................................................
47_1       ....................................................................................................
Consensus  FRDSARSSVTSGDSVTDYLARTRRAASEATGGLDRERIDLLWKIARAGARSAVGTEARRQFRYDGDMNIGVITDFELEVRNALNRRAHAVGAQDVVQHGT
                  310       320       330       340       350       360       370       380       390       400
           ----:----|----:----|----:----|----:----|----:----|----:----|----:----|----:----|----:----|----:----|
1_1        ..........................................................................F.........................
2_1        .....................................................................G..............................
3_1        .....................................................................G..............................
4_1        ..........................................................................F.........................
5_1        .....................................................................G..............................
```

6_1	....T....................................
7_1	....F.........................
8_1	....F.........................
9_1	....F.........................
10_1	....T....................................
11_1	....T......................S.............
12_1	....T......................S.............
13_1	....T.....I..............................
14_1	....T......................S.............
15_1	....T....................................
16_1	....T......................S.............
17_1	....G..............................
18_1	....T....................................
19_1	....T....................................
20_1	....G..............................
21_1	....G..............................
22_1	....T......................S.............
23_1	....
24_1	....
25_1	....G..............................
26_1	....
27_1	....G..............................
28_1	....T......................S.............
29_1	....
30_1	....G..............................
31_1	....T......................S.............
32_1	....T....................................
33_1	....T....................................
34_1	....T....................................
35_1	....T....................................
36_1	....T......................S.............
37_1	....G..............................
38_1	....
39_1	....G..............................

```
40_1      ...............................................................T....................................
41_1      .....................................................................I..............................
42_1      ..........................................................................F.........................
43_1      ..........................................................................F.........................
44_1      ..........................................................................F.........................
45_1      ....................................................................................................
46_1      .....................................................................G..............................
47_1      ..........................................................................F.........................
Consensus EQNNPFPEADEKIFVVSATGESQMLTRGQLKEYIGQQRGEGYVFYENRAYGVAGKSLFDDGLGAAPGVPSGRSKSSPDVLETVPASPGLRRPSLGAVERQ
                   410       420       430       440       450       460       470       480       490       500
          ----:----|----:----|----:----|----:----|----:----|----:----|----:----|----:----|----:----|----:----|
1_1       ....................................................................................................
2_1       ....................................................................................................
3_1       ....................................................................................................
4_1       ....................................................................................................
5_1       ....................................................................................................
6_1       ............................V.............P.............L....S.K.V..................................
7_1       ....................................................................................................
8_1       ....................................................................................................
```



```
9_1       ....................................................................................................
10_1      ...............................................................K...T................................
11_1      .........L......M.D.........V.............P.............L......K.V........................G.........
12_1      .........L........D.........V........V....P.............L......K.V........................G.........
13_1      ............................V.............P.............L....S.K.V..................................
14_1      .........L........D.........V........V....P.............L......K.V..A.....................G.........
15_1      .......G....................V.............P.............L....S.K.V........................A.........
16_1      .........L......M.D.........V.............P.............L......K.V........................G.........
17_1      ....................................................................................................
18_1      ...............................................................K...T................................
19_1      ...............................................................K...T................................
```

```
20_1       ....................................................................................................
21_1       ....................................................................................................
22_1       .........L......M.D.........V........V....P.............L....S.K.V..................................
23_1       ....................................................................................................
24_1       ...............................................................K...T................................
25_1       ....................................................................................................
26_1       ....................................................................................................
27_1       ....................................................................................................
28_1       .........L........D.........V........V....P.............L......K.V........................G.........
29_1       ....................................................................................................
30_1       ....................................................................................................
31_1       .........L......M.D.........V.............P.............L......K.V........................G.........
32_1       .......G....................V.............P.............L....S.K.V........................A.........
33_1       ............................V.............P.............L....S.K.V..................................
34_1       .......G....................V.............P.............L....S.K.V........................G.........
35_1       ............................V.............P.............L....S.K.V..................................
36_1       .........L........D.........V........V....P.............L......K.V........................G.........
37_1       .......H............................................................................................
38_1       ....................................................................................................
39_1       ....................................................................................................
40_1       ............................V.............P.............L....S.K.V..................................
41_1       ............................V.............P.............L....S.K.V..................................
42_1       ...........................................................................G........................
43_1       ....................................................................................................
44_1       ....................................................................................................
45_1       ....................................................................................................
46_1       ....................................................................................................
47_1       ....................................................................................................
Consensus  DSGYDSLDGVGSRSFSLGEVSDMAAVEAAELEMTRQVLHAGARQDDAEPGVSGASAHWGQRALQGAQAVAAAQRLVHAIALMTQFGRAGSTNTPQEAASL
                 510       520       530       540       550       560       570       580       590       600
           ----:----|----:----|----:----|----:----|----:----|----:----|----:----|----:----|----:----|----:----|
1_1        ....................................................................................................
2_1        ....................................................................................................
3_1        ..................................................................V.................................
```

```
4_1         ....................................................................................................
5_1         ..................................................................V.................................
6_1         ....................................................................................................
7_1         ....................................................................................................
8_1         ....................................................................................................
9_1         ....................................................................................................
10_1        .....................L..............................................................................
11_1        ....................................................................................................
12_1        ....................................................................................................
13_1        ....................................................................................................
14_1        ....................................................................................................
15_1        ....................................................................................................
16_1        ....................................................................................................
17_1        ....................................................................................................
18_1        .....................L..............................................................................
19_1        .....................L..............................................................................
20_1        ....................................................................................................
21_1        ....................................................................................................
22_1        ....................................................................................................
23_1        ....................................................................................................
24_1        .....................L..............................................................................
25_1        ....................................................................................................
26_1        ....................................................................................................
27_1        ....................................................................................................
28_1        ....................................................................................................
29_1        ....................................................................................................
30_1        ....................................................................................................
31_1        ....................................................................................................
32_1        ....................................................................................................
33_1        ....................................................................................................
34_1        ....................................................................................................
```

```
35_1      ....................................................................................................
36_1      ....................................................................................................
37_1      ...........................................................S........................................
38_1      ....................................................................................................
39_1      ............V.......................................................................................
40_1      ....................................................................................................
41_1      ....................................................................................................
42_1      ....................................................................................................
43_1      ....................................................................................................
44_1      ....................................................................................................
45_1      ....................................................................................................
46_1      ....................................................................................................
47_1      ....................................................................................................
Consensus SAAVFGLGEASSAVAETVSGFFRGSSRWAGGFGVAGGAMALGGGIAAAVGAGMSLTDDAPAGQKAAAGAEIALQLTGGTVELASSIALALAAARGVTSGL
                 610       620       630       640       650       660       670       680       690       700
          ----:----|----:----|----:----|----:----|----:----|----:----|----:----|----:----|----:----|----:----|
1_1       ....................................................................................................
2_1       ....................................................................................................
3_1       ....................................................................................................
4_1       ....................................................................................................
5_1       ....................................................................................................
6_1       ....................................................................................................
7_1       ....................................................................................................
8_1       ....................................................................................................
9_1       ....................................................................................................
10_1      .....................................................................................S..............
11_1      ....................................................................................................
12_1      ....................................................................................................
13_1      ....................................................................................................
14_1      ....................................................................................................
15_1      ....................................................................................................
16_1      .....A..............................................................................................
17_1      ....................................................................................................
18_1      .....................................................................................S..............
```

```
19_1      .....................................................................................S..............
20_1      ....................................................................................................
21_1      ....................................................................................................
22_1      ....................................................................................................
23_1      ....................................................................................................
24_1      .....................................................................................S..............
25_1      ....................................................................................................
26_1      ....................................................................................................
27_1      ....................................................................................................
28_1      ....................................................................................................
29_1      ........................................................................T...........................
30_1      ....................................................................................................
31_1      .....A..............................................................................................
32_1      ....................................................................................................
33_1      ....................................................................................................
34_1      ....................................................................................................
35_1      ....................................................................................................
36_1      .....A..............................................................................................
37_1      ....................................................................................................
38_1      ....................................................................................................
39_1      ....................................................................................................
40_1      ....................................................................................................
41_1      ....................................................................................................
42_1      ....................................................................................................
43_1      ....................................................................................................
44_1      ....................................................................................................
45_1      ........................................................................T...........................
46_1      ....................................................................................................
47_1      ....................................................................................................
Consensus QVAGASAGAAAGALAAALSPMEIYGLVQQSHYADQLDKLAQESSAYGYEGDALLAQLYRDKTAAEGAVAGVSAVLSTVGAAVSIAAAASVVGAPVAVVTS
                 710       720       730       740       750       760       770       780       790       800
          ----:----|----:----|----:----|----:----|----:----|----:----|----:----|----:----|----:----|----:----|
1_1       ....................................................................................................
2_1       ...................................................................................................A
```

```
3_1         ...................................................................................................A
4_1         ....................................................................................................
5_1         ...................................................................................................A
6_1         ....................................................................................................
7_1         ....................................................................................................
8_1         ....................................................................................................
9_1         ....................................................................................................
```



```
10_1        ...................................................................................................A
11_1        ....................................................................................................
12_1        ....................................................................................................
13_1        ....................................................................................................
14_1        ....................................................................................................
15_1        ....................................................................................................
16_1        ....................................................................................................
17_1        ...................................................................................................A
18_1        .........................................S..........................................................
19_1        .........................................S..........................................................
20_1        ...................................................................................................A
21_1        ...................................................................................................A
22_1        ....................................................................................................
23_1        ...................................................................................................A
24_1        .........................................S..........................................................
25_1        ...................................................................................................A
26_1        ...................................................................................................A
27_1        ...................................................................................................A
28_1        ....................................................................................................
29_1        ...................................................................................................A
30_1        ...................................................................................................A
31_1        ....................................................................................................
32_1        ....................................................................................................
```

```
33_1      ....................................................................................................
34_1      ....................................................................................................
35_1      ....................................................................................................
36_1      ....................................................................................................
37_1      ...................................................................................................A
38_1      ...................................................................................................A
39_1      ...................................................................................................A
40_1      ....................................................................................................
41_1      ....................................................................................................
42_1      ....................................................................................................
43_1      ....................................................................................................
44_1      ....................................................................................................
45_1      ...................................................................................................A
46_1      ...................................................................................................A
47_1      ....................................................................................................
Consensus LLTGALNGILRGVQQPIIEKLANDYARKIDELGGPQAYFEKNLQARHEQLANSDGLRKMLADLQAGWNASSVIGVQTTEISKSALELAAITGNADNLKSV
                 810       820       830       840       850       860       870       880       890       900
          ----:----|----:----|----:----|----:----|----:----|----:----|----:----|----:----|----:----|----:----|

1_1       ....................................................................................................
2_1       .......I............................................................................................
3_1       .......I............................................................................................
4_1       ....................................................................................................
5_1       .......I............................................................................................
6_1       ....................................................................................................
7_1       ....................................................................................................
8_1       ....................................................................................................
9_1       ...........................................................................................M........
10_1      .......I............................................................................................
11_1      ....................................................................................................
12_1      ....................................................................................................
13_1      ....................................................................................................
14_1      ....................................................................................................
15_1      ....................................................................................................
16_1      ....................................................................................................
```

```
17_1        .......I............................................................................................
18_1        .......I............................................................................................
19_1        .......I............................................................................................
20_1        .......I............................................................................................
21_1        .......I............................................................................................
22_1        ....................................................................................................
23_1        .......I............................................................................................
24_1        .......I............................................................................................
25_1        .......I............................................................................................
26_1        .......I............................................................................................
27_1        .......I............................................................................................
28_1        ....................................................................................................
29_1        .......I............................................................................................
30_1        .......I............................................................................................
31_1        ....................................................................................................
32_1        ....................................................................................................
33_1        ....................................................................................................
34_1        ....................................................................................................
35_1        ....................................................................................................
```



```
36_1        ....................................................................................................
37_1        .......I............................................................................................
38_1        .......I............................................................................................
39_1        .......I............................................................................................
40_1        ....................................................................................................
41_1        ....................................................................................................
42_1        ....................................................................................................
43_1        ....................................................................................................
44_1        ....................................................................................................
45_1        .......I............................................................................................
46_1        .......I............................................................................................
```

```
47_1      ....................................................................................................
Consensus DVFVDRFVQGERVAGQPVVLDVAAGGIDIASRKGERPALTFITPLAAPGEEQRRRTKTGKSEFTTFVEIVGKQDRWRIRDGAADTTIDLAKVVSQLVDAN
                 910       920       930       940       950       960       970       980       990      1000
          ----:----|----:----|----:----|----:----|----:----|----:----|----:----|----:----|----:----|----:----|
1_1       ....................................................................................................
2_1       .........E...................................................................K......................
3_1       .........E...................................................................K......................
4_1       ....................................................................................................
5_1       .........E...................................................................K......................
6_1       ....................................................................................................
7_1       ....................................................................................................
8_1       ....................................................................................................
9_1       ....................................................................................................
10_1      .....................................I...............E.......................K......................
11_1      ....................................................................................................
12_1      ....................................................................................................
13_1      ...........S........................................................................................
14_1      ....................................................................................................
15_1      ...........S........................................................................................
16_1      ....................................................................................................
17_1      .........E...................................................................K......................
18_1      .....................................I...............E.......................K......................
19_1      .....................................I...............E.......................K......................
20_1      .........E...................................................................K......................
21_1      .........E...................................................................K......................
22_1      ...........S........................................................................................
23_1      .........E...................................................................K......................
24_1      .....................................I...............E.......................K......................
25_1      .........E...................................................................K......................
26_1      .........E...................................................................K......................
27_1      .........E...................................................................K......................
28_1      ....................................................................................................
29_1      .........E...................................................................K......................
30_1      .........E...................................................................K......................
```

```
31_1      ....................................................................................................
32_1      ...........S........................................................................................
33_1      ...........S........................................................................................
34_1      ....................................................................................................
35_1      ....................................................................................................
36_1      ....................................................................................................
37_1      .............................................................................K......................
38_1      .........E...................................................................K......................
39_1      .........E...................................................................K......................
40_1      ....................................................................................................
41_1      ...........S........................................................................................
42_1      ....................................................................................................
43_1      ....................................................................................................
44_1      ....................................................................................................
45_1      .........E...................................................................K......................
46_1      .........E...................................................................K......................
47_1      ....................................................................................................
Consensus GVLKHSIKLDVIGGDGDDVVLANASRIHYDGGAGTNTVSYAALGRQDSITVSADGERFNVRKQLNNANVYREGVATQTTAYGKRTENVQYRHVELARVGQ
                   1010      1020      1030      1040      1050      1060      1070      1080      1090      1100
          ----:----|----:----|----:----|----:----|----:----|----:----|----:----|----:----|----:----|----:----|
1_1       V.....................................................Q.............................................
2_1       ..................................A...................H...V.........................................
3_1       ..................................A...................H...V.........................................
4_1       ......................................................Q.............................................
5_1       ..................................A...................H...V.........................................
6_1       ......................................................Q.............................................
7_1       ......................................................Q.............................................
8_1       ......................................................Q.............................................
9_1       ......................................................Q.............................................
10_1      ..................................A..............A....H.............................................
```

```
11_1        ......................................................Q.............................................
12_1        ......................................................Q.............................................
13_1        ......................................................Q.............................................
14_1        ......................................................Q.............................................
15_1        ......................................................Q.............................................
16_1        ......................................................Q.............................................
17_1        ..................................A...................H...V.........................................
18_1        ..................................A..............A....H.............................................
19_1        ..................................A..............A....H.............................................
20_1        ..................................A...................H...V.........................................
21_1        ..................................A...................H...V.........................................
22_1        ......................................................Q.............................................
23_1        ..................................A...................H...V.........................................
24_1        ..................................A..............A....H.............................................
25_1        ..................................A...................H...V.........................................
26_1        ..................................A...................H...V.........................................
27_1        ..................................A...................H...V.........................................
28_1        ......................................................Q.............................................
29_1        ..................................A...................H...V.........................................
30_1        ..................................A...................H...V.........................................
31_1        ......................................................Q...............................L.............
32_1        ......................................................Q.............................................
33_1        ......................................................Q.............................................
34_1        ......................................................Q.............................................
35_1        ......................................................Q.............................................
36_1        ......................................................Q.............................................
37_1        ..................................A...................H...V...................N.....................
38_1        ..................................A...................H...V.........................................
39_1        ..................................A...................H...V.........................................
40_1        ......................................................Q.............................................
41_1        ......................................................Q.............................................
42_1        ......................................................Q.............................................
43_1        ................G.....................................Q.............................................
44_1        ......................................................Q.............................................
```

```
45_1       ..................................A...................H...V.........................................
46_1       ..................................A...................H...V.........................................
47_1       ......................................................Q.............................................
Consensus  LVEVDTLEHVQHIIGGAGNDSITGNAHDNFLAGGSGDDRLDGGAGNDTLVGGEGxNTVIGGAGDDVFLQDLGVWSNQLDGGAGVDTVKYNVHQPSEERLE
                    1110      1120      1130      1140      1150      1160      1170      1180      1190      1200
           ----:----|----:----|----:----|----:----|----:----|----:----|----:----|----:----|----:----|----:----|
1_1        .................................................Q..................................................
2_1        .......................................S...S............D......H....................................
3_1        .......................................S...S............D......H....................................
4_1        .................................................Q..................................................
5_1        ......................................TS...S............D......H....................................
6_1        ....................................................................................................
7_1        .................................................Q..................................................
8_1        .................................................Q..................................................
9_1        .................................................Q..................................................
10_1       ............N...........L..............S...S............D...........................................
11_1       .............................................V......................................................
12_1       .............................................V......................................................
13_1       ............Q.......................................................................................
14_1       .............................................V......................................................
15_1       .................................................Q..................................................
16_1       .............................................V...................W..................................
17_1       ...E...................................S...S............D......H....................................
18_1       ............N...........L..............S...S............D...........................................
19_1       ........................L..............S...S............D......H....................................
20_1       .......................................S...S............D......H...................D................
21_1       ...E...................................S...S............D......H....................................
22_1       .............................................V......................................................
23_1       ...E...................................S...S............D......H....................................
24_1       ............N...........L..............S...S............D...........................................
25_1       .......................................S...S............D......H....................................
26_1       ...E...................................S...S............D......H....................................
27_1       ...E...................................S...S............D......H....................................
28_1       .............................................V......................................................
```

```
29_1      ...E...................................S...S............D......H....................................
30_1      ...E...................................S...S............D......H....................................
31_1      .................................................Q..................................................
32_1      ....................................................................................................
33_1      ....................................................................................................
34_1      .............................................V......................................................
35_1      ....................................................................................................
36_1      .............................................V......................................................
```



```
37_1      .......................................S...S............D......H....................................
38_1      .......................................S...S............D......H....................................
39_1      ...E...................................S...S............D......H....................................
40_1      ....................................................................................................
41_1      ............Q.......................................................................................
42_1      .................................................Q..................................................
43_1      .................................................Q..................................................
44_1      .................................................Q..................................................
45_1      ...E...................................S...S............D......H....................................
46_1      ...E...................................S...S............D......H....................................
47_1      .................................................Q..................................................
Consensus RMGDTGIHADLQKGTVEKWPALNLFSVDHVKNIENLHGSRLNDRIAGDDRDNELWGHDGNDTIRGRGGDDILRGGLGLDTLYGEDGNDIFLQDDETVSDD
                1210      1220      1230      1240      1250      1260      1270      1280      1290      1300
          ----:----|----:----|----:----|----:----|----:----|----:----|----:----|----:----|----:----|----:----|
1_1       ....................................................................................................
2_1       .................A.K................EG.....ARR.M....S..S............................................
3_1       .................A.K................EG.....ARR.MG...S..S............................................
4_1       ....................................................................................................
5_1       .................A.K................EG.....ARR.MG...S..S............................................
6_1       ....................................................................................................
7_1       ....................................................................................................
8_1       ....................................................................................................
```

```
9_1     .....................................................................................................
10_1    .................A.K................................S................................................
11_1    .....................................................................................................
12_1    ....................................................S.......................................D........
13_1    .....................................................................................................
14_1    ....................................................S.......................................D........
15_1    .....................................................................................................
16_1    ....................................................S................................................
17_1    .................A.K................................S................................................
18_1    .................A.K................................S................................................
19_1    .................A.K................................S................................................
20_1    ......Q.....T.L..A.E................EG.....ARR.M....S..S.............................................
21_1    .................A.K................................S................................................
22_1    .....................................................................................................
23_1    .................A.K................................S................................................
24_1    .................A.K................................S................................................
25_1    .................A.K................EG.....ARR.M....S..S.............................................
26_1    .................A.K................................S................................................
27_1    .................A.K................................S................................................
28_1    .....................................................................................................
29_1    .................A.K................................S................................................
30_1    .................A.K................................S................................................
31_1    .....................................................................................................
32_1    .....................................................................................................
33_1    .....................................................................................................
34_1    .....................................................................................................
35_1    .....................................................................................................
36_1    .....................................................................................................
37_1    ............T.L..A.E................EG.....ARR.M....S..S.............................................
38_1    .................A.K................................S................................................
39_1    .................A.K................................S................................................
40_1    .....................................................................................................
41_1    .....................................................................................................
42_1    .....................................................................................................
```

```
43_1       ....................................................................................................
44_1       .........................................................................K..........................
45_1       .................A.K................................S...............................................
46_1       .................A.K................................S...............................................
47_1       ....................................................................................................
Consensus  IDGGAGLDTVDYSAMIHPGRIVAPHEYGFGIEADLSREWVRKASALGVDYYDNVRNVENVIGTSMKDVLIGDAQANTLMGQGGDDTVRGGDGDDLLFGGD
                   1310      1320      1330      1340      1350      1360      1370      1380      1390      1400
           ----:----|----:----|----:----|----:----|----:----|----:----|----:----|----:----|----:----|----:----|
1_1        ...................................Q................................................................
2_1        ...................................P..........A.........A.....V.T...................................
3_1        ...................................P..........A.........A.....V.T...................................
4_1        ...................................Q................................................................
5_1        ...................................P..........A.........A.....V.T...................................
6_1        ...................................Q................................................................
7_1        ...................................Q................................................................
8_1        ...................................Q................................................................
9_1        ...................................Q................................................................
10_1       ...................................P..........A.........A.....V.T...................................
11_1       ...................................Q................................................................
```



```
12_1       ...................................Q..................................................T.............
13_1       ...................................Q................................................................
14_1       ...................................Q..................................................T.............
15_1       ...................................Q..................................................T.............
16_1       ...................................Q..........A.....................................................
17_1       ...................................P..........A.........A.....V.T...................................
18_1       ...................................P..........A.........A.....V.T...................................
19_1       ...................................P..........A.........A.....V.T...................................
20_1       ...................................P..........A.........A.....V.T...................................
21_1       ...................................P..........A.........A.....V.T...................................
22_1       ...................................Q................................................................
```

```
23_1       ...................................P..........A.........A.....V.T...................................
24_1       ...................................P..........A.........A.....V.T...................................
25_1       ...................................P..........A.........A.....V.T...................................
26_1       ...................................P..........A.........A.....V.T...................................
27_1       ...................................P..........A.........A.....V.T...................................
28_1       ...................................Q................................................................
29_1       ...................................P..........A.........A.....V.T...................................
30_1       ...................................P..........A.........A.....V.T...................................
31_1       ...................................Q..................................................T.............
32_1       ...................................Q................................................................
33_1       ...................................Q..........A.......................................T.............
34_1       ...................................Q..................................................T.............
35_1       ...................................Q................................................................
36_1       ...................................Q................................................................
37_1       ...................................P..........A.........A.....V.T...................................
38_1       ...................................P..........A.........A.....V.T...................................
39_1       ...................................P..........A.........A.....V.T...................................
40_1       ...................................Q................................................................
41_1       ...................................P................................................................
42_1       ...................................Q................................................................
43_1       ...................................Q................................................................
44_1       ...................................Q................................................................
45_1       ...................................P..........A.........A.....V.T...................................
46_1       ...................................P..........A.........A.....V.T...................................
47_1       ...................................Q................................................................
Consensus  GNDMLYGDAGNDTLYGGLGDDTLEGGAGNDWFGQTxAREHDVLRGGDGVDTVDYSQTGAHAGIAAGRIGLGILADLGAGRVDKLGEAGSSAYDTVSGIEN
                 1410      1420      1430      1440      1450      1460      1470      1480      1490      1500
           ----:----|----:----|----:----|----:----|----:----|----:----|----:----|----:----|----:----|----:----|
1_1        ....................................................................................................
2_1        ................................................................................N...................
3_1        ........................................E.......................................N...................
4_1        ....................................................................................................
5_1        ........................................E.......................................N...................
6_1        ....................................................................................................
```

```
7_1     ....................................................................................................
8_1     ....................................................................................................
9_1     ....................................................................................................
10_1    ................................................................................N...................
11_1    ....................................................................................................
12_1    ....................................................................................................
13_1    ....................................................................................................
14_1    ....................................................................................................
15_1    ....................................................................................................
16_1    ............................................................N.......................................
17_1    ........................................E.......................................N...................
18_1    ................................................................................N...................
19_1    ........................................E.......................................N...................
20_1    ........................................E.......................................N...................
21_1    ........................................E.......................................N...................
22_1    ....................................................................................................
23_1    ........................................E.......................................N...................
24_1    ................................................................................N...................
25_1    ................................................................................N...................
26_1    ........................................E.......................................N...................
27_1    ........................................E.......................................N...................
28_1    ....................................................................................................
29_1    ........................................E.......................................N...................
30_1    ........................................E.......................................N...................
31_1    ....................................................................................................
32_1    ....................................................................................................
33_1    ....................................................................................................
34_1    ....................................................................................................
35_1    ....................................................................................................
36_1    ....................................................................................................
37_1    ........................................E.......................................N...................
```

```
38_1      ........................................E.......................................N...................
39_1      ........................................E.......................................N...................
40_1      ..............................................................................G.....................
41_1      ....................................................................................................
42_1      ....................................................................................................
43_1      ....................................................................................................
44_1      ....................................................................................................
45_1      ........................................E.......................................N...................
46_1      ........................................E.......................................N...................
47_1      ....................................................................................................
Consensus VVGTELADRITGDAQANVLRGAGGADVLAGGEGDDVLLGGDGDDQLSGDAGRDRLYGEAGDDWFFQDAANAGNLLDGGDGRDTVDFSGPGRGLDAGAKGV
                1510      1520      1530      1540      1550      1560      1570      1580      1590      1600
          ----:----|----:----|----:----|----:----|----:----|----:----|----:----|----:----|----:----|----:----|
1_1       ......................N.............................................................................
2_1       ..............................V..............................................................A......
3_1       ..............................V..............................................................A......
4_1       ......................N.............................................................................
5_1       ..............................V..............................................................A......
6_1       ....................................................................................................
7_1       ......................N.............................................................................
8_1       ......................N.............................................................................
9_1       ......................N.............................................................................
10_1      ..............................V..............................................................A......
11_1      ....................................................................................................
12_1      ......................N.............................................................................
13_1      ....................................................................................................
14_1      ......................N.............................................................................
15_1      ....................................................................................................
16_1      ....................................................................................................
17_1      ..............................V..............................................................A......
18_1      ..............................V..............................................................A......
19_1      ..............................V..............................................................A......
20_1      ..............................V..............................................................A......
21_1      ..............................V..............................................................A......
```

```
22_1       ....................................................................................................
23_1       ..............................V..............................................................A......
24_1       ..............................V..............................................................A......
25_1       ..............................V..............................................................A......
26_1       ..............................V.................T............................................A......
27_1       ..............................V..............................................................A......
28_1       ....................................................................................................
29_1       ..............................V..............................................................A......
30_1       ..............................V..............................................................A......
31_1       ....................................................................................................
32_1       ....................................................................................................
33_1       ......................N.............................................................................
34_1       ....................................................................................................
35_1       ....................................................................................................
36_1       ....................................................................................................
37_1       ..............................V..............................................................A......
38_1       ..............................V..............................................................A......
39_1       ..............................V.................T............................................A......
40_1       ...................L................................................................................
41_1       ....................................................................................................
42_1       ......................N.............................................................................
43_1       ......................N.............................................................................
44_1       ......................N.............................................................................
45_1       ..............................V..............................................................A......
46_1       ..............................V..............................................................A......
47_1       ......................N.............................................................................
Consensus  FLSLGKGFASLMDEPETSNVLRHIENAVGSARDDVLIGDAGANVLNGLAGNDVLSGGAGDDVLLGDEGSDLLSGDAGNDDLFGGQGDDTYLFGVGYGHDT
                 1610      1620      1630      1640      1650      1660      1670      1680      1690      1700
           ----:----|----:----|----:----|----:----|----:----|----:----|----:----|----:----|----:----|----:----|
1_1        ......................................................I.........Q...................................
2_1        ..............................................................I.....................................
3_1        ..............................................................I.....................................
4_1        ......................................................I.........Q...................................
5_1        ..............................................................I.....................................
```

```
6_1       ....................................................................................................
7_1       ......................................................I.........Q...................................
8_1       ......................................................I.........Q...................................
9_1       ......................................................I.........Q...................................
10_1      .............................................................T......................................
11_1      ....................................................................................................
12_1      ....................................................................................................
```



```
13_1      ....................................................................................................
14_1      ....................................................................................................
15_1      ....................................................................................................
16_1      ....................................................................................................
17_1      ..............................................................I.....................................
18_1      .............................................................T......................................
19_1      ..............................................................I.....................................
20_1      ..............................................................I.....................................
21_1      ..............................................................I.....................................
22_1      ....................................................................................................
23_1      ..............................................................I.....................................
24_1      .............................................................T......................................
25_1      ..............................................................I.....................................
26_1      ..............................................................I.....................................
27_1      ..............................................................I.....................................
28_1      ....................................................................................................
29_1      ..............................................................I.....................................
30_1      ..............................................................I.....................................
31_1      ........................R...........................................................................
32_1      ....................................................................................................
33_1      ....................................................................................................
34_1      ....................................................................................................
35_1      ....................................................................................................
```

```
36_1      ....................................................................................................
37_1      ..............................................................I...........................P.........
38_1      ..............................................................I.....................................
39_1      ..............................................................I.....................................
40_1      ....................................................................................................
41_1      ....................................................................................................
42_1      ......................................................I.........Q...................................
43_1      ......................................................I.........Q...................................
44_1      ......................................................I.........Q...................................
45_1      ..............................................................I.....................................
46_1      ..............................................................I.....................................
47_1      ......................................................I.........Q...................................
Consensus IYESGGGHDTIRINAGADQLWFARQGNDLEIRILGTDDALTVHDWYRDADHRVEAIHAANQAVDPAGIEKLVEAMAQYPDPGAAAAAPPAARVPDTLMQS
          ----:-
1_1       ......
2_1       ......
3_1       ......
4_1       ......
5_1       ......
6_1       ......
7_1       ......
8_1       ......
9_1       ......
10_1      ......
11_1      ......
12_1      ......
13_1      ......
14_1      ......
15_1      ......
16_1      ......
17_1      ......
18_1      ......
19_1      ......
20_1      ......
```

```
21_1       ......
22_1       ......
23_1       ......
24_1       ......
25_1       ......
26_1       ......
27_1       ......
28_1       ......
29_1       ......
30_1       ......
31_1       ......
32_1       ......
33_1       ......
34_1       ......
35_1       ......
36_1       ......
37_1       ......
38_1       ......
```



```
39_1       ......
40_1       ......
41_1       ......
42_1       ......
43_1       ......
44_1       ......
45_1       ......
46_1       ......
47_1       ......
Consensus LAVNWR
```
