# Supplementary material for: Characterization of Post-Translational Modifications and Cytotoxic Properties of the Adenylate-Cyclase Hemolysin Produced by Various Bordetella pertussis and Bordetella parapertussis Isolates
Source: Toxins (Basel). 2017 Sep 26;9(10):304. doi: 10.3390/toxins9100304 (PMC5666351; doi:10.3390/toxins9100304)
Supplement: Supplementary file 1 [file toxins-09-00304-s001.zip › FigureS2.pdf]

Bp K-860 Myristoylation – Best MSMS Spectra – FR5388

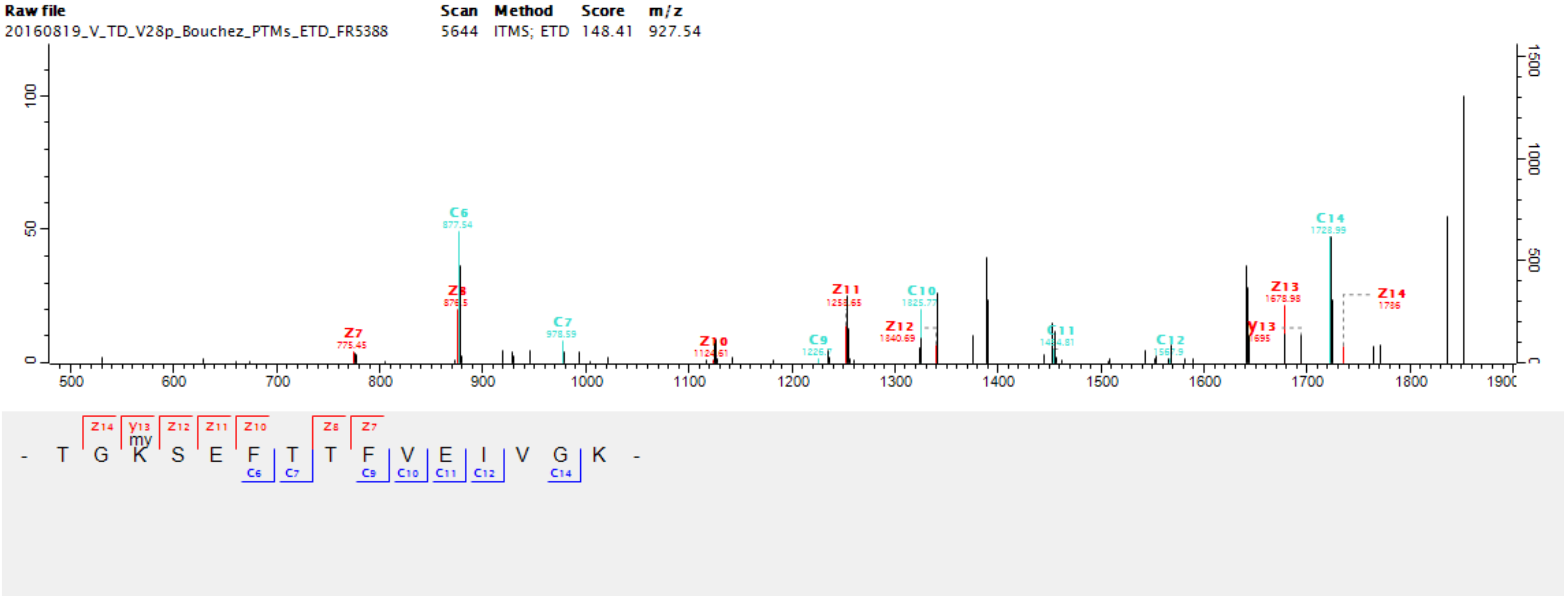

MS/MS spectrum of the myristoylated lysine K-860 of Bp-AC-Hly
Annotated MaxQuant MS/MS spectrum corresponding to the peptide containing the modified Lysine K-860 with a myristoyl group.

Bp K-860 Palmitoylation – Best MSMS Spectra – FR5388

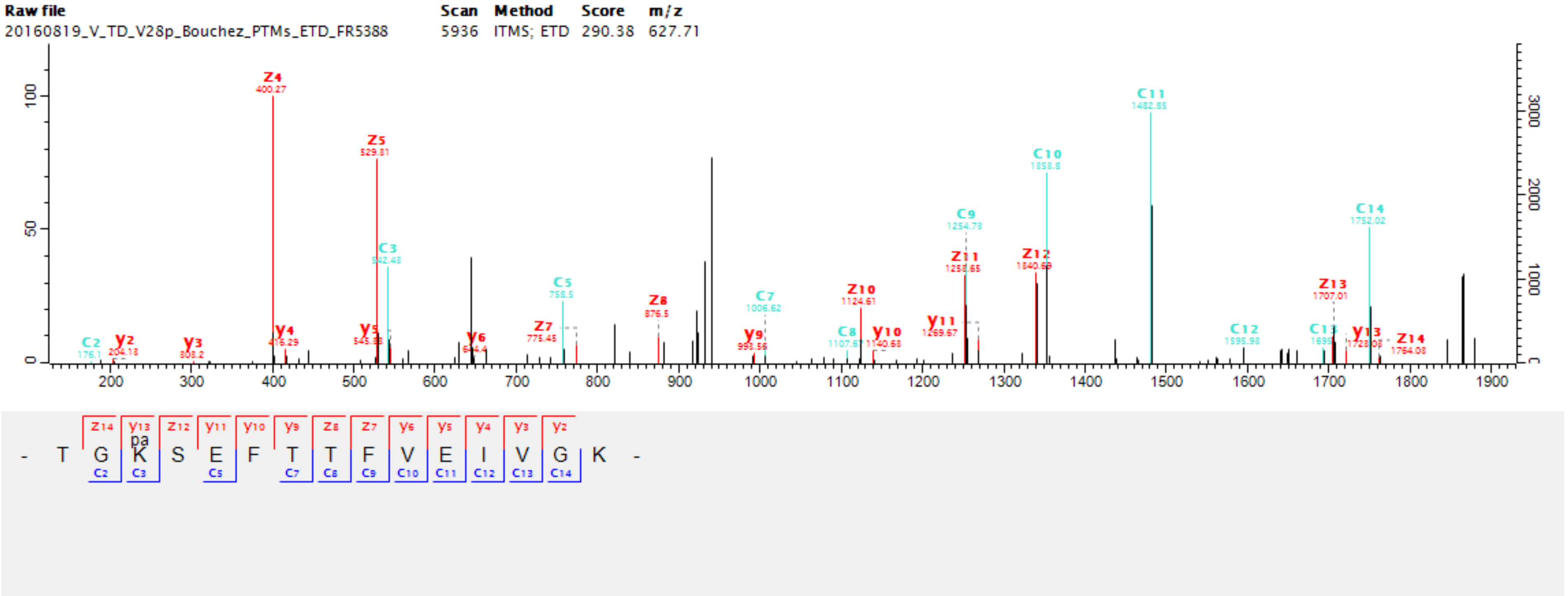

MS/MS spectrum of the palmitoylated lysine K-860 of Bp-AC-Hly

Annotated MaxQuant MS/MS spectrum corresponding to the peptide containing the modified Lysine K-860 with a palmitoyl group.

Bp K-860 Palmitoylation – MSMS Spectra – T8132

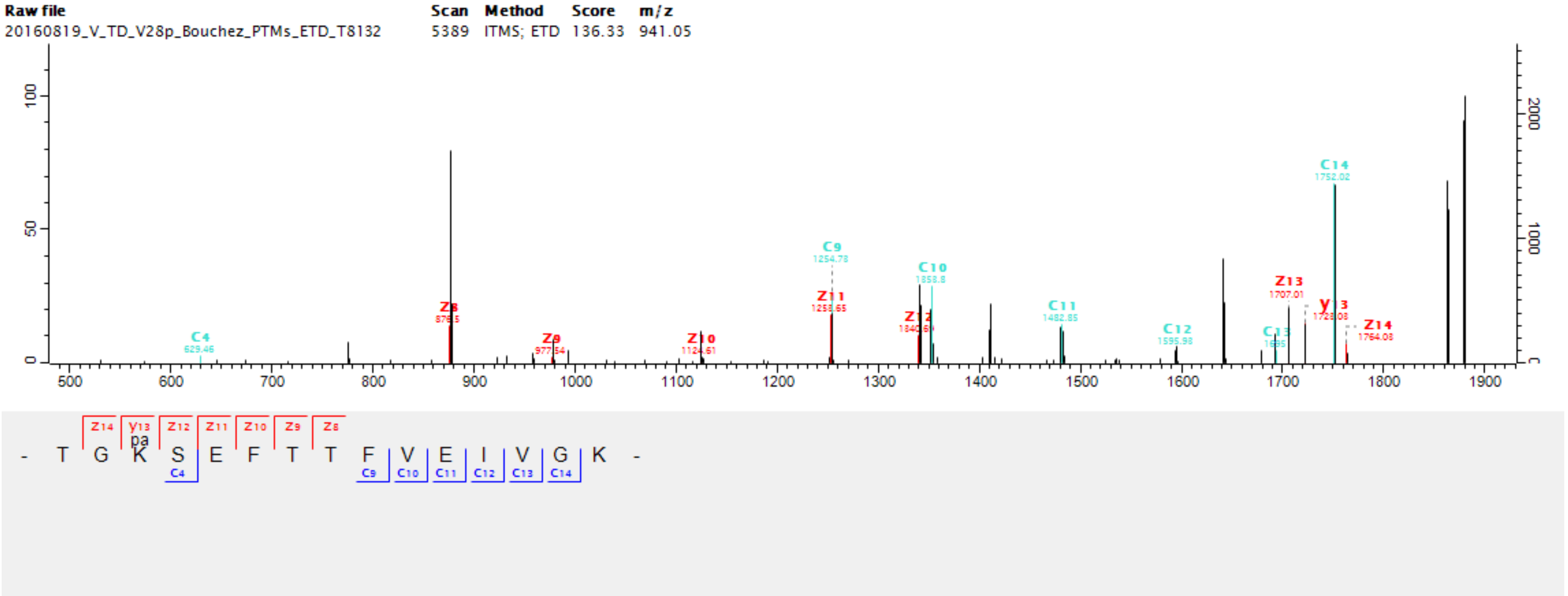

MS/MS spectrum of the palmitoylated lysine K-860 of Bp-AC-Hly

Annotated MaxQuant MS/MS spectrum corresponding to the peptide containing the modified Lysine K-860 with a palmitoyl group.

Bp K-860 Unmodified – Best MSMS Spectra – T8132

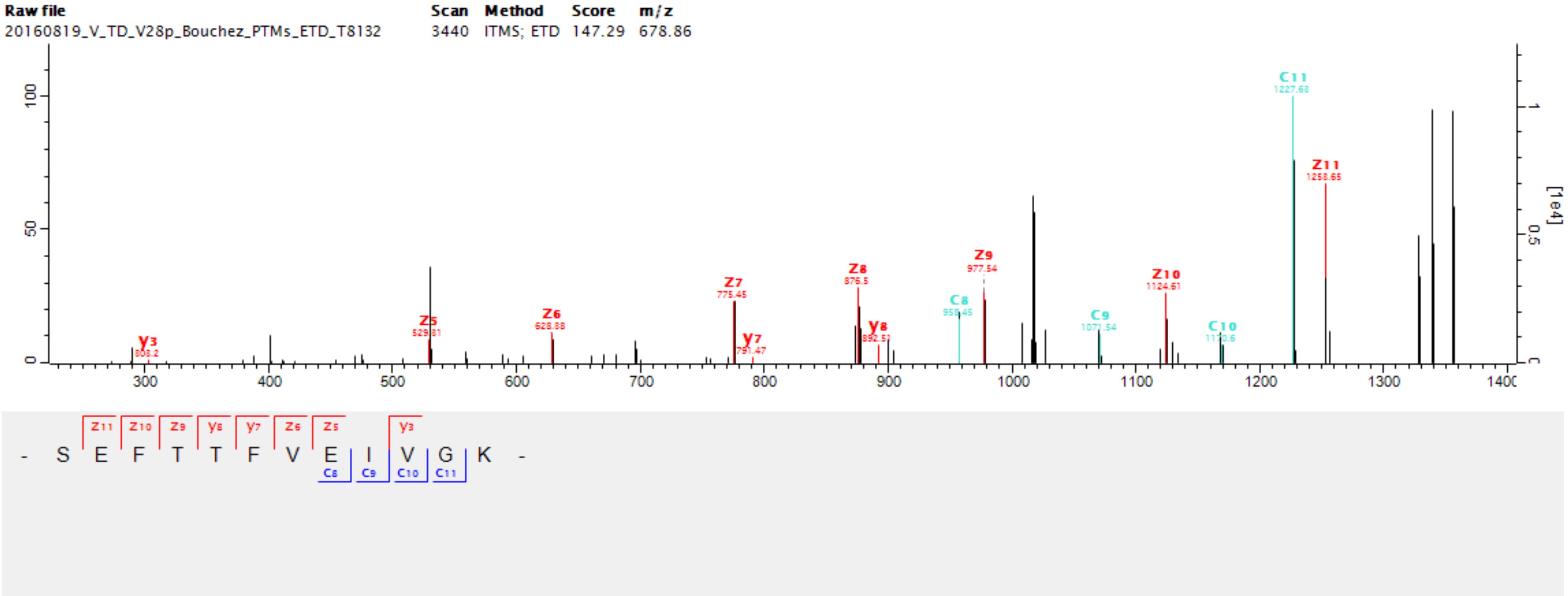

MS/MS spectrum of the unmodified lysine K-860 of Bp-AC-Hly

Annotated MaxQuant MS/MS spectrum corresponding to the peptide containing the unmodified Lysine K-860.

Bp K-983 Myristoylation – Best MSMS Spectra – FR4624

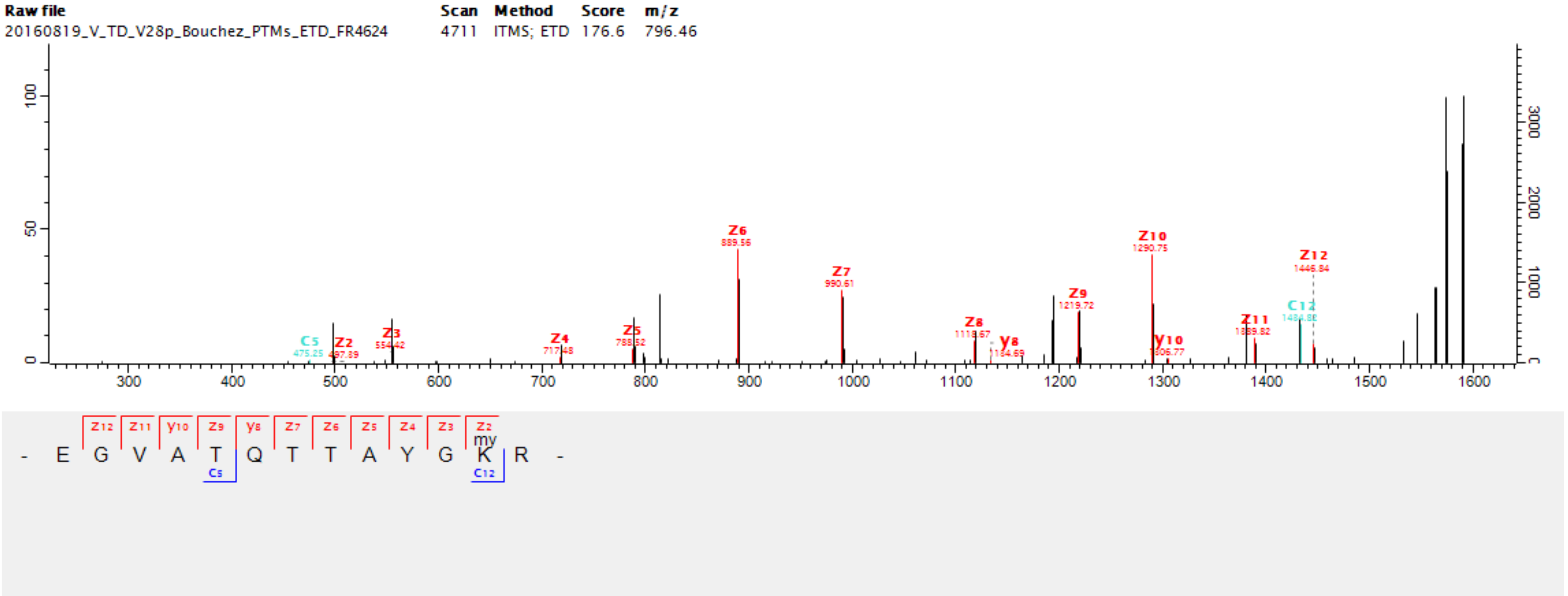

MS/MS spectrum of the myristoylated lysine K-983 of Bp-AC-Hly

Annotated MaxQuant MS/MS spectrum corresponding to the peptide containing the modified Lysine K-983 with a myristoyl group.

Bp K-983 Myristoylation – MSMS Spectra – T8132

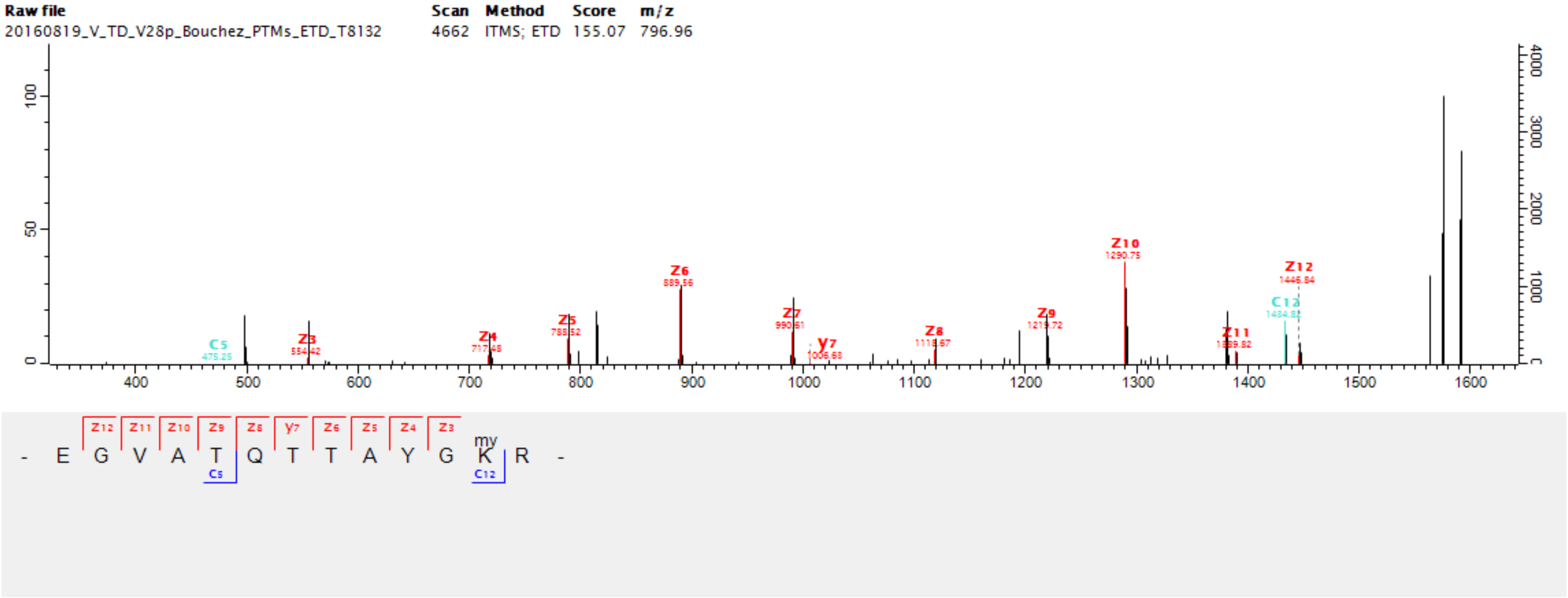

MS/MS spectrum of the myristoylated lysine K-983 of Bp-AC-Hly

Annotated MaxQuant MS/MS spectrum corresponding to the peptide containing the modified Lysine K-983 with a myristoyl group.

Bp K-983 Palmitoylation – Best MSMS Spectra – FR5388

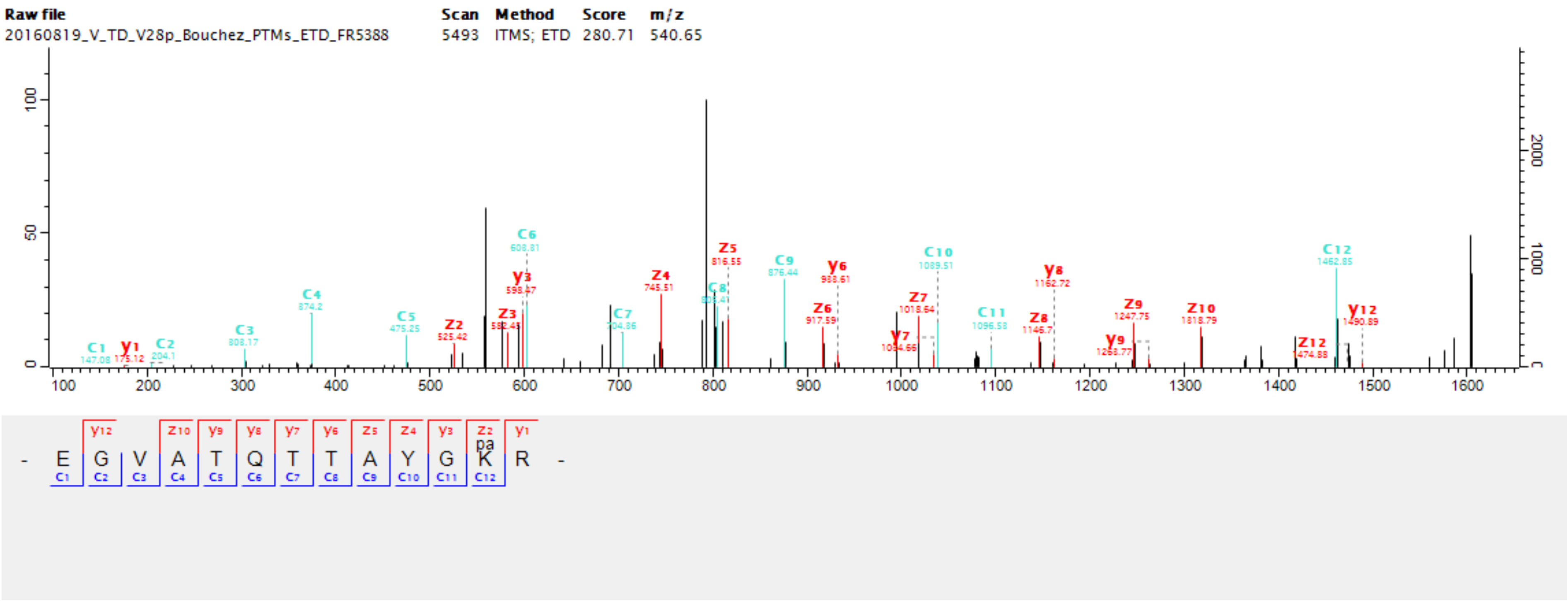

MS/MS spectrum of the palmitoylated lysine K-983 of Bp-AC-Hly

Annotated MaxQuant MS/MS spectrum corresponding to the peptide containing the modified Lysine K-983 with a palmitoyl group.

Bp K-983 Palmitoylation – MSMS Spectra – T8132

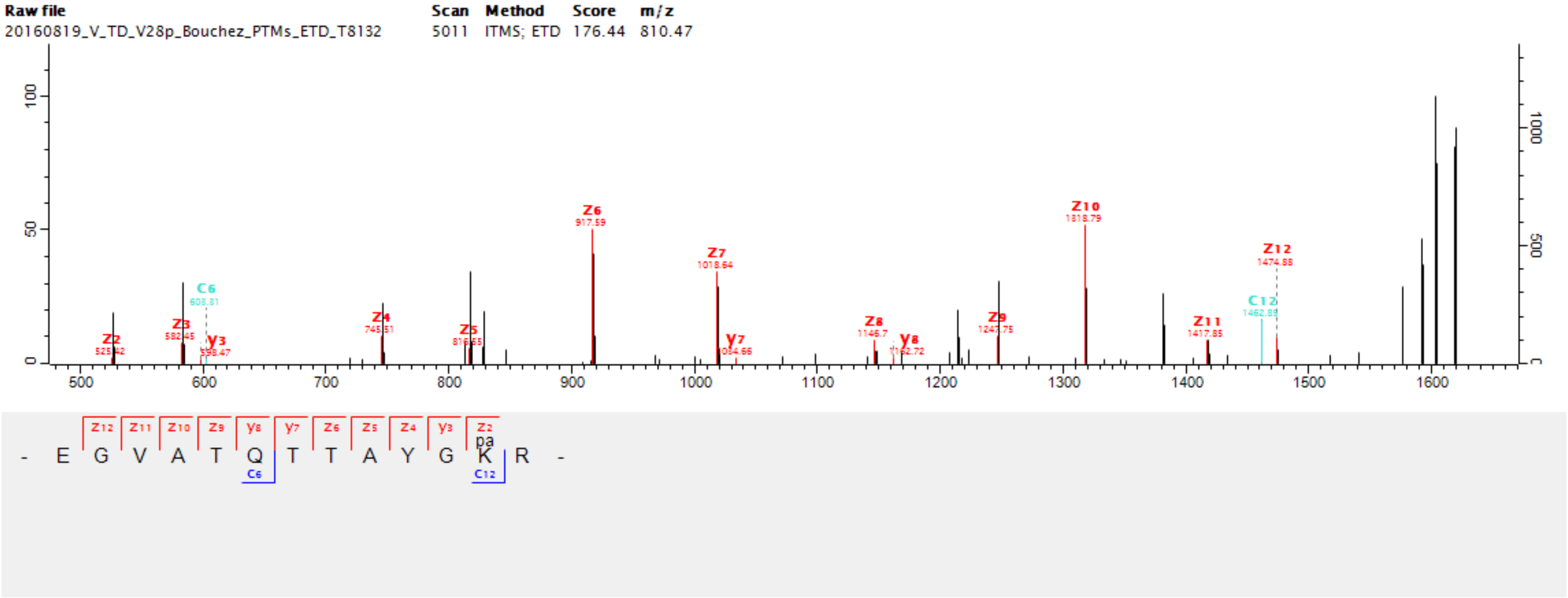

MS/MS spectrum of the palmitoylated lysine K-983 of Bp-AC-Hly

Annotated MaxQuant MS/MS spectrum corresponding to the peptide containing the modified Lysine K-983 with a palmitoyl group.
