# Supplementary material for: Characterization of Post-Translational Modifications and Cytotoxic Properties of the Adenylate-Cyclase Hemolysin Produced by Various Bordetella pertussis and Bordetella parapertussis Isolates
Source: Toxins (Basel). 2017 Sep 26;9(10):304. doi: 10.3390/toxins9100304 (PMC5666351; doi:10.3390/toxins9100304)
Supplement: Supplementary file 1 [file toxins-09-00304-s001.zip › FigureS3.pdf]

Bpp K-894 Myristoylation – Best MSMS Spectra – FR5340

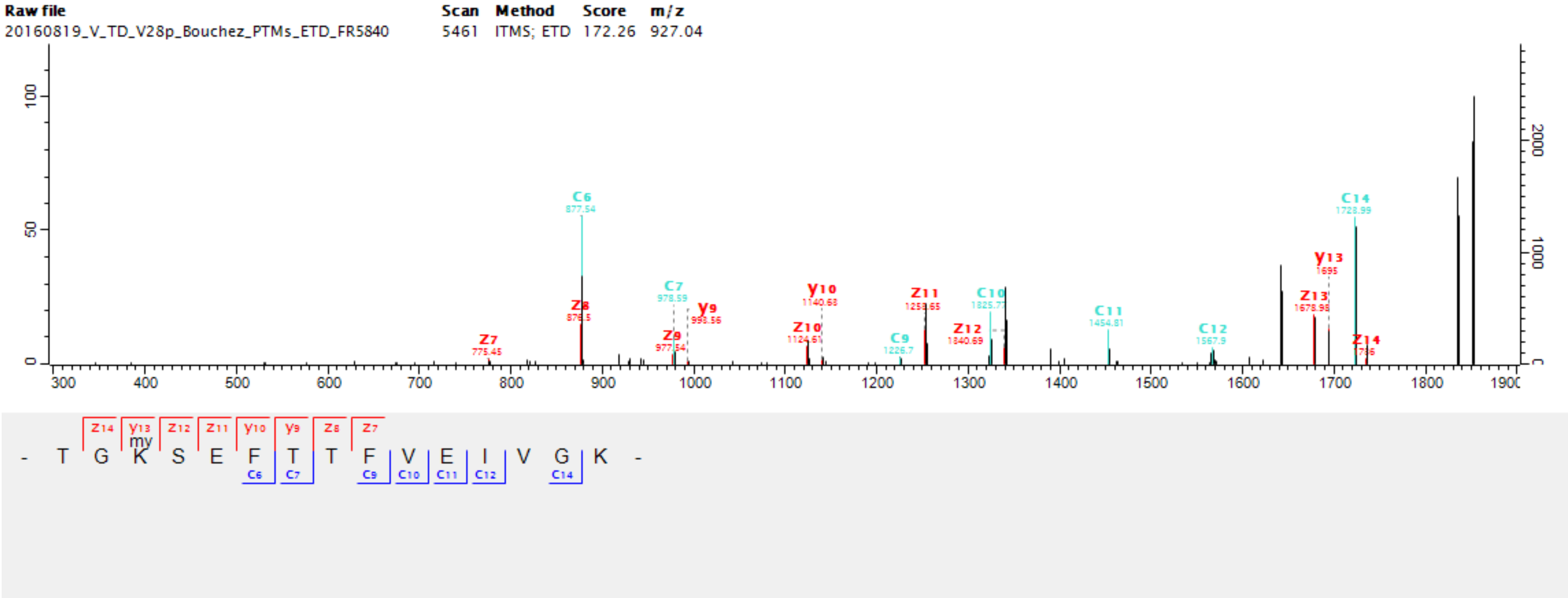

MS/MS spectrum of the myristoylated lysine K-894 of Bpp-AC-Hly

Annotated MaxQuant MS/MS spectrum corresponding to the peptide containing the modified Lysine K-894 with a myristoyl group.

Bpp K-894 Myristoylation – MSMS Spectra – 12822

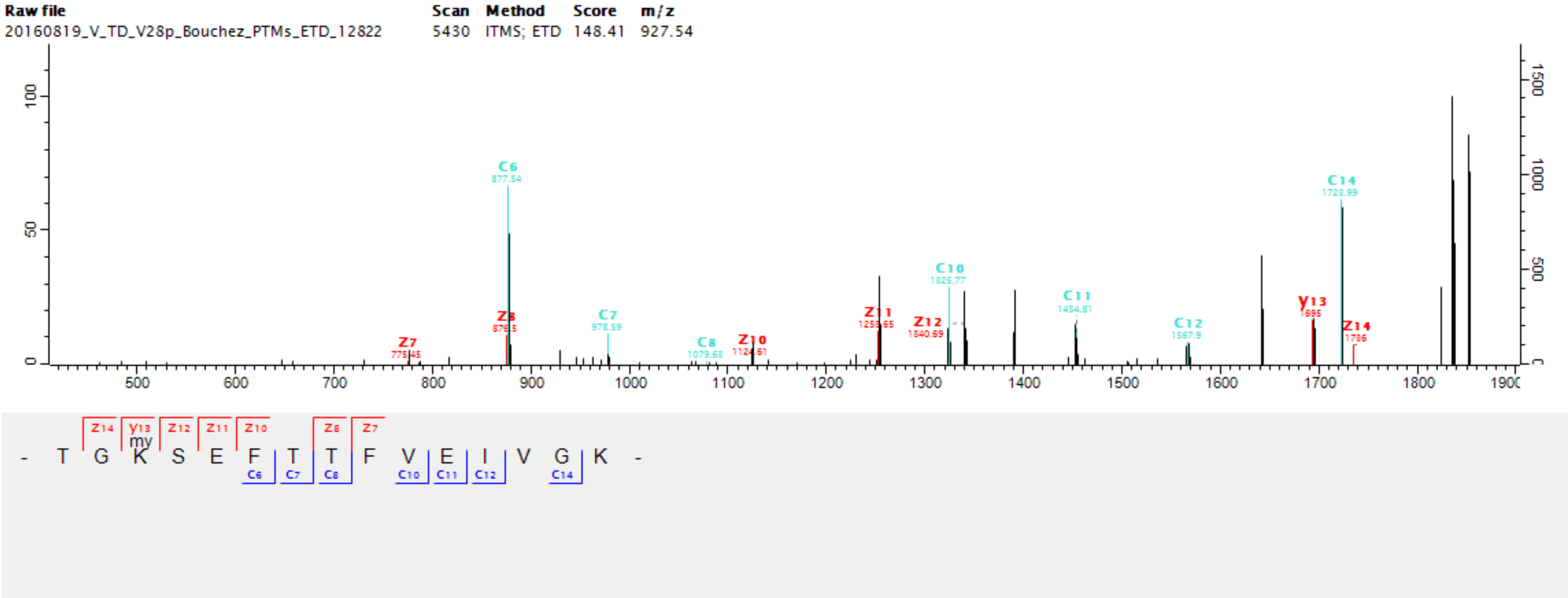

MS/MS spectrum of the myristoylated lysine K-894 of Bpp-AC-Hly

Annotated MaxQuant MS/MS spectrum corresponding to the peptide containing the modified Lysine K-894 with a myristoyl group.

Bpp K-894 Palmitoylation – Best MSMS Spectra – FR3728

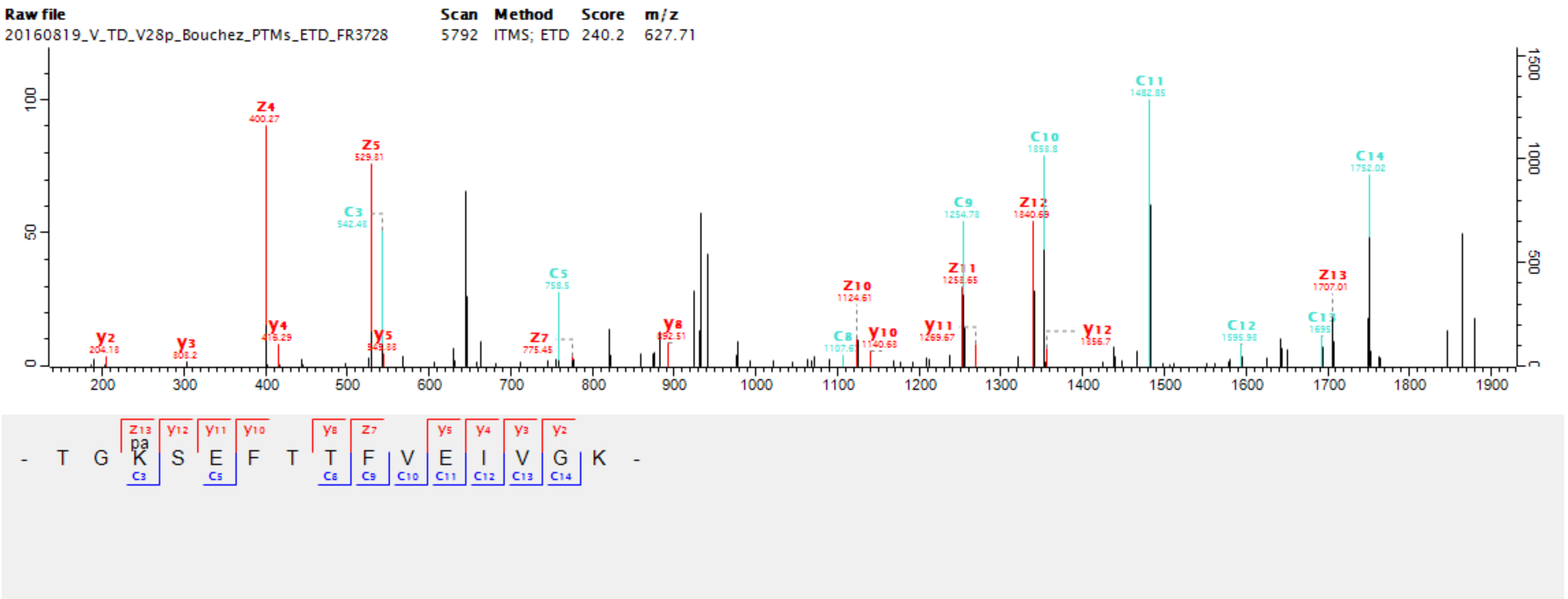

MS/MS spectrum of the palmitoylated lysine K-894 of Bpp-AC-Hly

Annotated MaxQuant MS/MS spectrum corresponding to the peptide containing the modified Lysine K-894 with a palmitoyl group.

Bpp K-894 Palmitoylation – MSMS Spectra – 12822

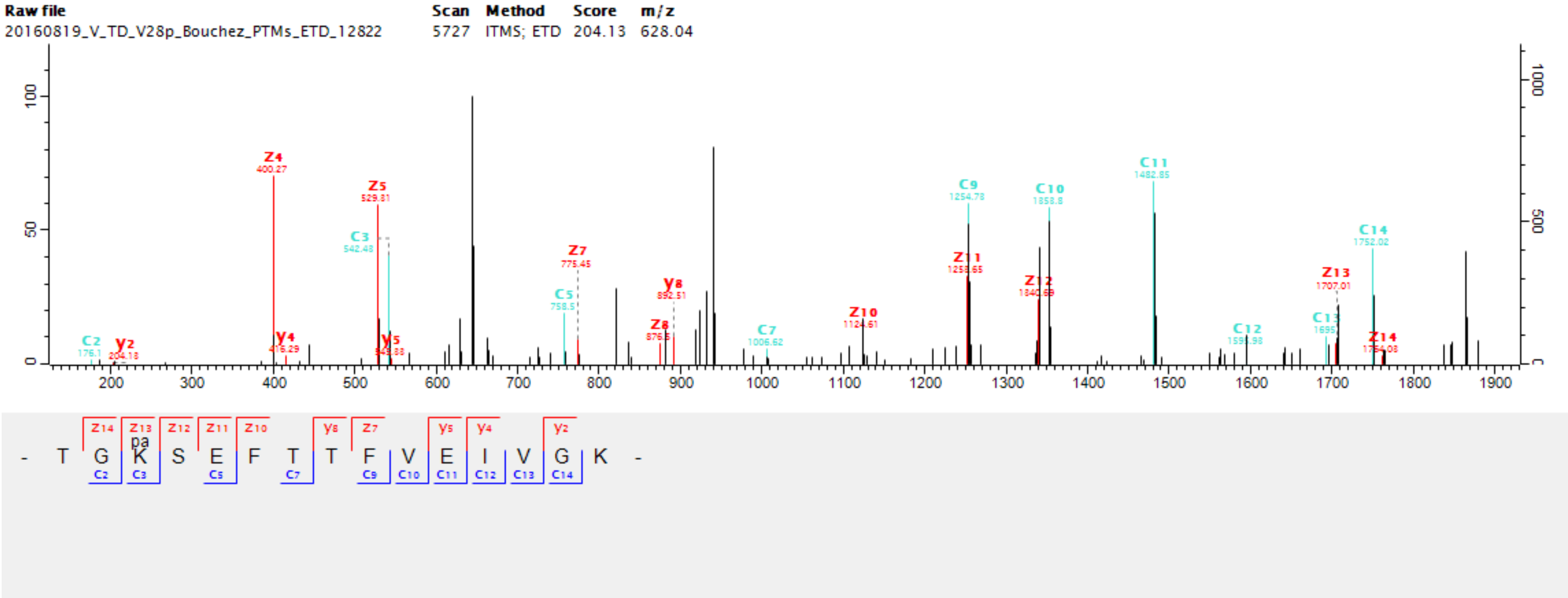

MS/MS spectrum of the palmitoylated lysine K-894 of Bpp-AC-Hly

Annotated MaxQuant MS/MS spectrum corresponding to the peptide containing the modified Lysine K-894 with a palmitoyl group.

Bpp K-1017 Myristoylation – Best MSMS Spectra – 12822

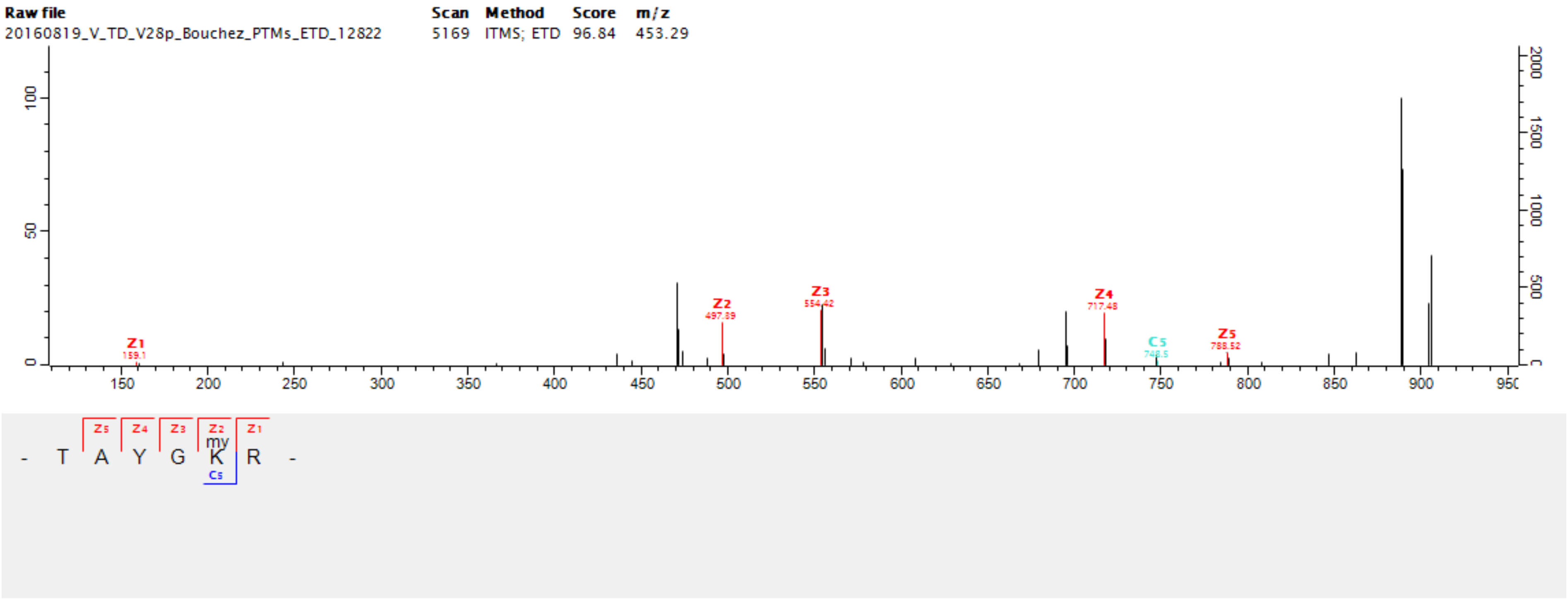

MS/MS spectrum of the myristoylated lysine K-1017 of Bpp-AC-Hly
Annotated MaxQuant MS/MS spectrum corresponding to the peptide containing the modified Lysine K-1017 with a myristoyl group.

Bpp K-1017 Palmitoylation – Best MSMS Spectra – BPP1

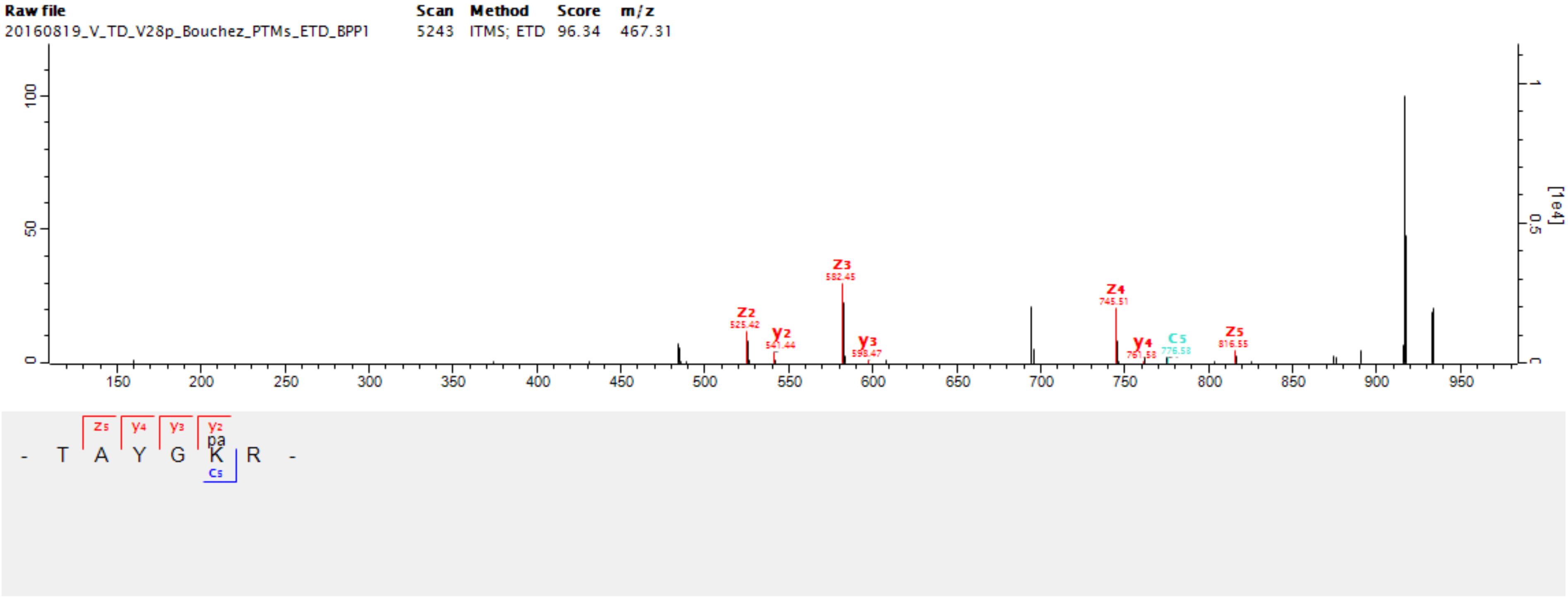

MS/MS spectrum of the palmitoylated lysine K-1017 of Bpp-AC-Hly

Annotated MaxQuant MS/MS spectrum corresponding to the peptide containing the modified Lysine K-1017 with a palmitoyl group.

Bpp K-1017 Palmitoylation – MSMS Spectra – 12822

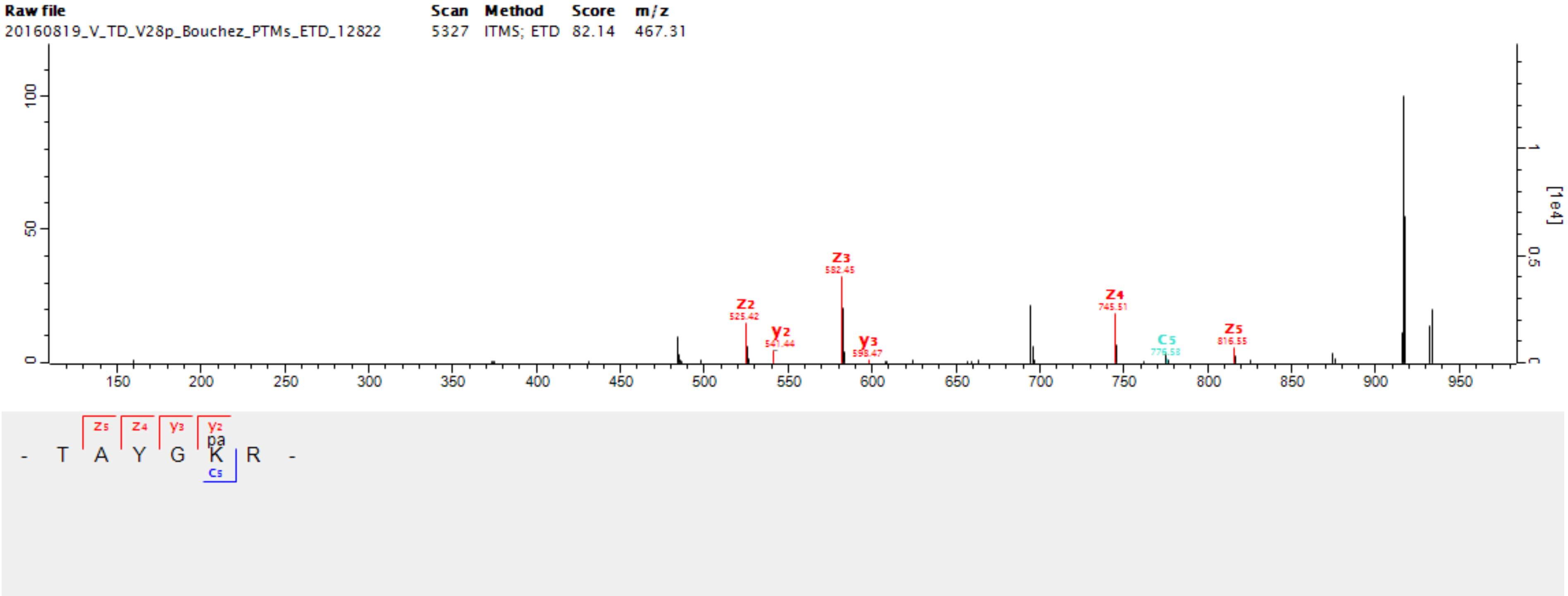

MS/MS spectrum of the palmitoylated lysine K-1017 of Bpp-AC-Hly

Annotated MaxQuant MS/MS spectrum corresponding to the peptide containing the modified Lysine K-1017 with a palmitoyl group.

Bpp K-1017 Palmitoleylation – Best MSMS Spectra – BPP1

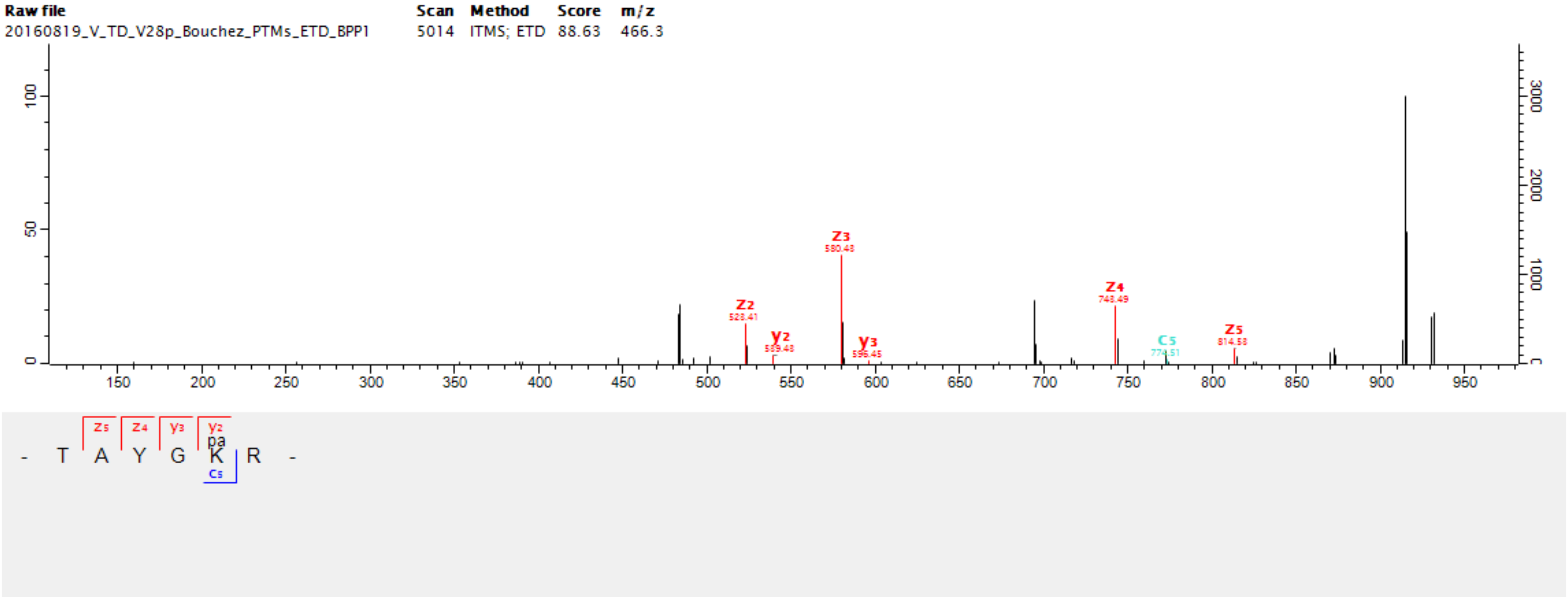

MS/MS spectrum of the palmitoleylated lysine K-1017 of Bpp-AC-Hly

Annotated MaxQuant MS/MS spectrum corresponding to the peptide containing the modified Lysine K-1017 with a palmitoleyl group.

Bpp K-1017 Palmitoleylation – MSMS Spectra – 12822

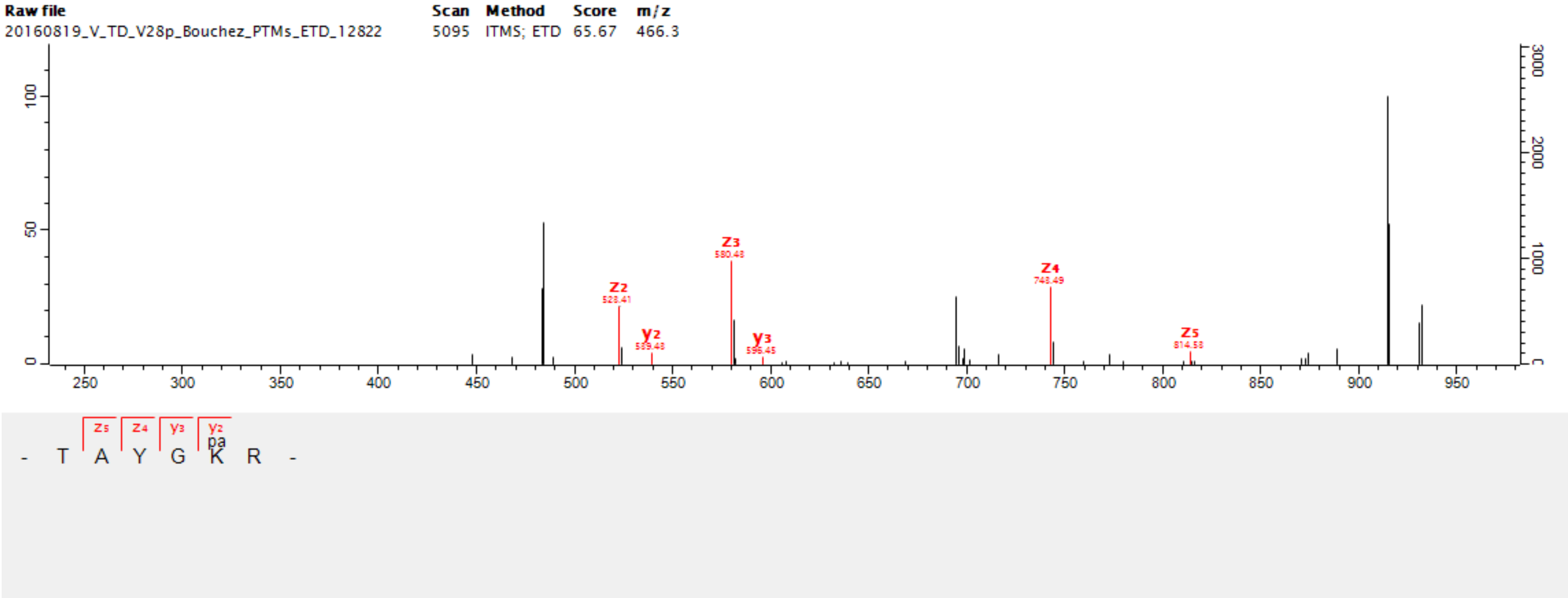

MS/MS spectrum of the palmitoleylated lysine K-1017 of Bpp-AC-Hly

Annotated MaxQuant MS/MS spectrum corresponding to the peptide containing the modified Lysine K-1017 with a palmitoleyl group.
