# Supplementary figures and images for: Characterization of Post-Translational Modifications and Cytotoxic Properties of the Adenylate-Cyclase Hemolysin Produced by Various Bordetella pertussis and Bordetella parapertussis Isolates
Source: Toxins (Basel). 2017 Sep 26;9(10):304. doi: 10.3390/toxins9100304 (PMC5666351; doi:10.3390/toxins9100304)

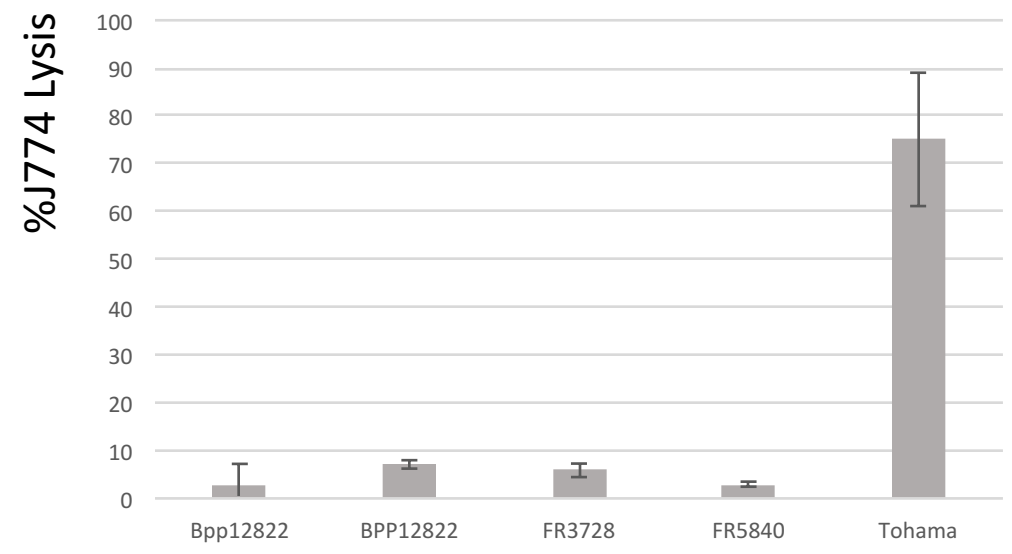

Supplement: Supplementary file 1 [file toxins-09-00304-s001.zip › FigureS4.pdf]
